# Molecular characterization and phylogenetics of Fennoscandian cowpox virus isolates based on the *p4c* and *atip* genes

**DOI:** 10.1186/1743-422X-11-119

**Published:** 2014-06-27

**Authors:** Malachy Ifeanyi Okeke, Arinze Stanley Okoli, Øivind Nilssen, Ugo Moens, Morten Tryland, Thomas Bøhn, Terje Traavik

**Affiliations:** 1 GenØk-Centre for Biosafety, The Science Park, PB 6418, Tromsø N-9294, Norway; 2 Institute of Medical Biology, Faculty of Health Sciences, UiT The Arctic University of Norway, Tromsø N-9037, Norway; 3 Department of Clinical Medicine, Medical Genetics, Faculty of Health Sciences, UiT The Arctic University of Norway, Tromsø N-9037, Norway; 4 Univerisity Hospital of North Norway, Tromsø N-9038, Norway; 5 Department of Arctic and Marine Biology, Faculty of Biosciences, Fisheries and Economics, UiT The Arctic University of Norway, Tromsø N-9037, Norway; 6 Institute of Pharmacy, Faculty of Health Sciences, UiT The Arctic University of Norway, Tromsø N-9037, Norway

**Keywords:** Cowpox virus, Orthopoxvirus, Phylogeny, Fennoscandia, atip, p4c

## Abstract

**Background:**

*Cowpox virus* (CPXV), a rodent-borne *Orthopoxvirus* (OPV) that is indigenous to Eurasia can infect humans, cattle, felidae and other animals. Molecular characterization of CPXVs isolated from different geographic locations is important for the understanding of their biology, geographic distribution, classification and evolution. Our aim was to characterize CPXVs isolated from Fennoscandia on the basis of A-type inclusion (ATI) phenotype, restriction fragment length polymorphism (RFLP) profiles of *atip* gene fragment amplicon, and phylogenetic tree topology in conjunction with the patristic and genetic distances based on full length DNA sequence of the *atip* and *p4c* genes.

**Methods:**

ATI phenotypes were determined by transmission electron microcopy and RFLP profiles were obtained by restriction enzyme digestion of the *atip* gene fragment PCR product. A 6.2 kbp region spanning the entire *atip* and *p4c* genes of Fennoscandian CPXV isolates was amplified and sequenced. The phylogenetic affinity of Fennoscandian CPXV isolates to OPVs isolated from other geographic regions was determined on the basis of the *atip* and *p4c* genes.

**Results:**

Fennoscandian CPXV isolates encoded full length *atip* and *p4c* genes. They produce wild type V^+^ ATI except for CPXV-No-H2. CPXVs were resolved into six and seven species clusters based on the phylogeny of the *atip* and *p4c* genes respectively. The CPXVs isolated from Fennoscandia were grouped into three distinct clusters that corresponded to isolates from Norway, Sweden and Finland.

**Conclusion:**

CPXV is a polyphyletic assemblage of six or seven distinct clusters and the current classification in which CPXVs are united as one single species should be re-considered. Our results are of significance to the classification and evolution of OPVs.

## Background

The *Poxviridae* is a family of large DNA viruses that multiplies in the cytoplasm of infected cells [1]. The *Poxviridae* family members are classified into two sub-families based on the hosts they infect [1]; viruses belonging to *Chordopoxvirinae* infect vertebrates whereas members of *Entomopoxvirinae* infect insects. The *Chordopoxvirinae* consists of at least eight genera and the *Orthopoxvirus* (OPV) is the most studied genus of the chordopoxviruses. OPVs are broadly divided into “Old World” and “North American” species. Members of the Old World OPVs include *Variola virus* (VARV), the etiologic agent of smallpox; *Vaccinia virus* (VACV), the vaccine virus used to eradicate smallpox; *Cowpox virus* (CPXV), a rodent- borne zoonotic OPV that is indigenous to Eurasia; *Monkeypox virus* (MPXV), a zoonotic OPV that causes smallpox-like diseases in humans; *Ectromelia virus* (ECTV), the etiologic agent of mousepox (lab mice); *Camelpox virus* (CMLV) and *Taterapox virus* (TATV) [2]. OPV species presumed to be endemic to North America include *Raccoonpox virus* (RCNV), *Volepox virus* (VPXV), and *Skunkpox virus* (SKPV) [1,3].

With the eradication and subsequent cessation of vaccination against smallpox, other OPVs especially MPXV in Central/West Africa, CPXV in Europe, VACV in Brazil and the Indian subcontinent are staging a comeback [4-11]. There is an increasing incidence of CPXV infections in humans, domestic cats, zoo animals and wild life [12,13]. Although human cowpox virus infection is in general characterized by mild and self-limiting lesions, they can also be fatal [14,15] especially in immune-compromised individuals. Molecular characterization of CPXVs isolated from different geographic locations is important in understanding their geographic distribution, variability and evolution, as well as monitoring the emergence of atypical CPXV strains with enhanced virulence. Such highly virulent CPXVs may arise due to (i) adaptive mutations in rodent or human host, (ii) recombination with other naturally occurring OPVs, (iii) recombination with genetically engineered OPVs or (iv) accidental escape of a lethal recombinant CPXV from laboratory containment. Recently, a CPXV cluster whose genomes are closer to CMLV, TATV and VARV has been identified [16]. In addition, the molecular characterization of CPXVs from different geographic regions will provide a baseline for assessing the potential for recombination between poxvirus-vectored vaccines and naturally circulating OPVs in regions where the former will be used to vaccinate against human, pets, production animals and wild-life diseases.

CPXVs are genetically heterogeneous [17,18]. They also show marked differences in phenotypic properties [19]. A recent phylogenetic analysis of the genome of 12 CPXVs shows that CPXV is a polyphyletic assemblage with at least five clades [20]. Although this study is the most comprehensive to date, it included only two isolates from Fennoscandia. The under-representation of the Fennoscandian isolates biased the assignment of the Norwegian human CPXV isolate to the same clade as strains isolated in the United Kingdom. The inclusion of more isolates from Scandinavia would help to clarify the phylogenetic position of Scandinavian CPXVs in relation to other OPVs. In order to study the evolutionary relationship between CPXVs and other OPVs, we sequenced the region spanning the entire *p4c* and *atip* open reading frames (ORFs) of CPXV isolated from specific Fennoscandian geographical locations and determined the phylogenetic relationships between these CPXVs and other OPVs on the basis of these sequences. The *atip* gene encodes the A-type inclusion protein (ATIP) [21,22] while the *p4c* encodes a protein that is required [23] but not sufficient [24,25] for the formation of wild type V^+^ A-type inclusion (ATI). It has been demonstrated that functionally intact *atip*, *p4c* and VACV Copenhagen *A27L* homologue are required for the formation of wild type V^+^ ATI [25]. The *atip* and *p4c* genes were selected because of the genetic stability being located in the central region of the genome, their high degree of conservation, their role in production of ATI, an inclusion body with a significant role in host to host transmission and viral survival outside the host [26], as well as history of use as a marker for differentiating OPVs. Both the *p4c* and *atip* genes are located in the central part of the genome and it is preferable to perform evolutionary analysis of OPV relatedness or divergence based on genes located in the central genome region, since genes located in the terminal region are highly variable due to adaptive selection and recombination [17,27,28]. Previously, amplification of an *atip* gene fragment with specific primers in conjunction with RFLP generated by restriction enzyme digestion of the *atip* gene fragment amplicon have been used to differentiate OPV species [29,30].

In this study, we investigated the sequence diversity and phylogeny of Fennoscandian CPXVs based on the *atip* and *p4c* genes. CPXVs isolated from Fennoscandia encoded intact *atip* and *p4c* ORFs. They also produced wild type V^+^ ATI except for CPXV-No-H2. Phylogenetically, CPXVs were resolved into six and seven distinct clusters based on the *atip* and *p4c* genes respectively. Fennoscandian CPXVs were segregated into three of the distinct clusters and isolates from one Fennoscandian country have closer phylogenetic relationship to each other than isolates from another country or geographical region.

## Results

### Fennoscandian CPXV isolates produce wild type V^+^ A-type inclusions

**Figure 1:**
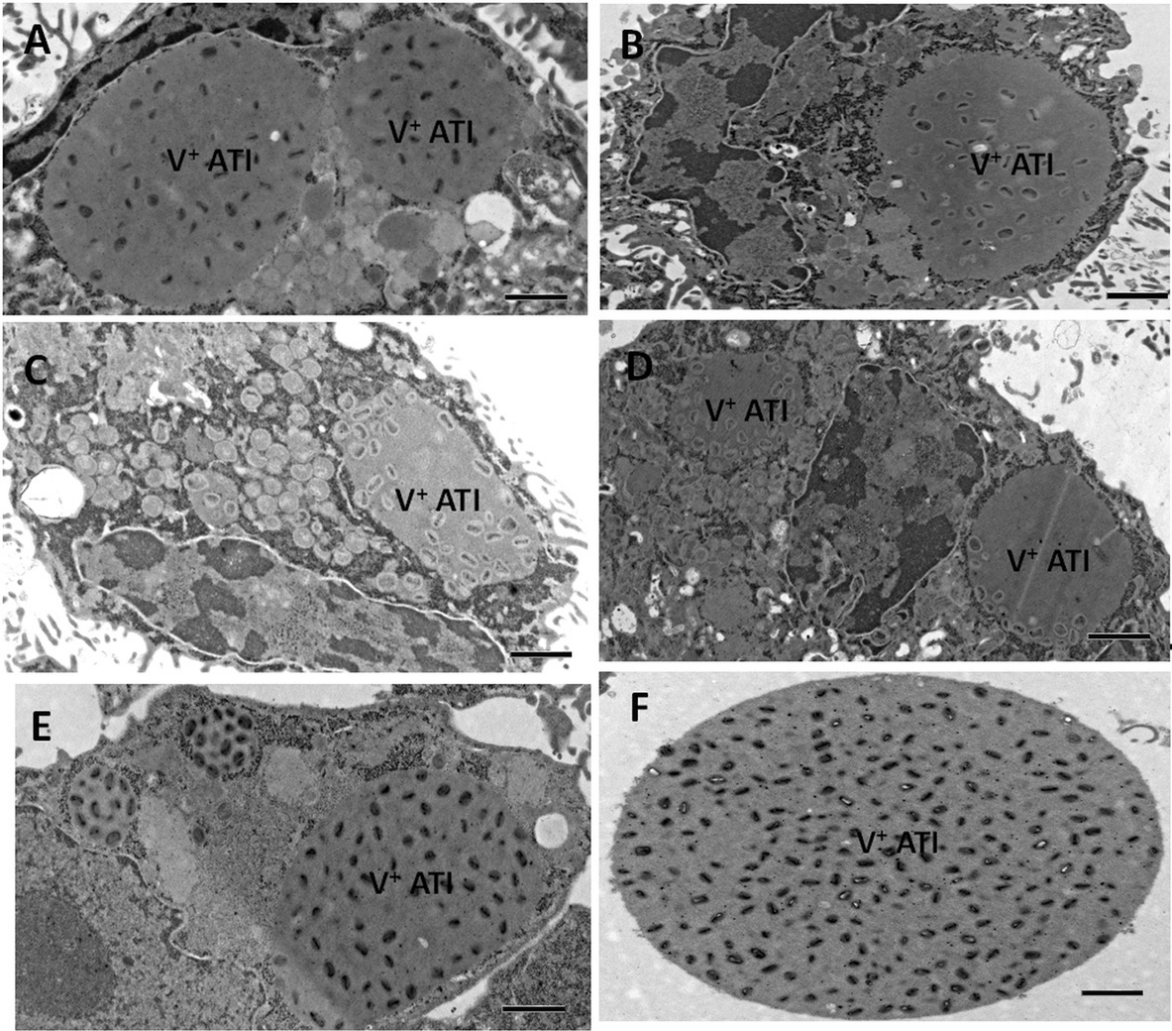
**The V**^**+ **^**A-type inclusion (ATI) produced by Fennoscandian CPXVs in infected Vero cells at 36 hours post infection.** Vero cells were infected with Fennoscandian CPXVs and CPXV-BR and processed for electron microscopy as described in Methods. **(A)** CPXV-No-F1, **(B)** CPXV-No-F2, **(C)** CPXV-Swe-H1, **(D)** CPXV-Swe-H2, **(E)** CPXV-FIN/T2000, **(F)** Cell free V^+^ATI from CPXV-FIN/T2000 infected Vero cells. The same ATI phenotype was produced in A549 cells (data not shown). Electron micrographs of the V^+^ATI produced in CPXV-No-H1 infected cells and the V^+/^ ATI produced in CPXV-No-H2 infected cells have been published previously [24,33]. Bars: 5 μm **(A-F)**.

The production of ATI was one of the first biological properties used to differentiate CPXV from VACV [31]. Among OPVs, only CPXV, ECTV and RCNV are known to produce ATI in infected cells [22,32,33]. Three strain specific ATI phenotypes are known to exist; the V^+^ ATI in which virions are within the ATI matrix, the V^−^ phenotype in which the ATI lacks virions within or on the surface, and the V^+/^ phenotype in which the ATIs lack internalized virions but are encrusted with virions on their surface [24,32,33]. To determine the ATI phenotype produced in cells infected with Fennoscandian CPXVs, we quantified ATI phenotypes in 50 cell sections that were clearly infected. Absolute and relative amounts of ATI phenotypes produced by each virus strain at 36 hours post infection were determined in infected Vero and A549 cells. Our results show that all the Fennoscandian CPXVs except CPXV-No-H2 produced the wild type V^+^ ATI in both Vero and A549 cells (Table 1, Figure 1). In Vero cells, the V^+^ATI ranged from 76.5% in CPXV-Swe-H1 to 98.6% in CPXV-FIN/T2000, while in A549 cells, the V^+^ ATI accounted for 93.9% - 100% of all ATI phenotype produced for each Fennoscandian CPXV strain (Table 1). The reason why more ATIs are produced in A549 cells compared to Vero was not investigated but may be related to the severity of cytopathic effects (CPE) in the respective cell lines. Fennoscandian CPXVs (except CPXV-FIN/T2000) were more cytopathic in Vero than in A549 cells (data not shown). Presumably, the reduced CPE in A549 leaves cells virtually intact allowing virion morphogenesis (including ATI production) to proceed optimally. The V^+^ ATI is very stable and remains intact even after cell lysis (Figure 1F).

**Table 1:** Phenotypes of A-type inclusion (ATI) in virus infected Vero and A549 cells

	**Actual and relative abundance of ATI at 36 hours post infection**^**a**^						
**Virus strain**	**Vero**			**A549**			**Source**
	**V**^**+**^	**V**^**+/**^	**V**^**−**^	**V**^**+**^	**V**^**+/**^	**V**^**−**^	
CPXV-No-H1	88 (93.6)	3 ( 3.2)	3 (3.2)	122 (97.6)	3 (2.4)	0 (0.0)	[33]
CPXV-No-F1	79 (84.9)	13 (14.0)	1 (1.1)	113 (94.9)	4 (3.4)	2 (1.7)	This study
CPXV-No-F2	71 (88.7)	4 (5.0)	5 (6.3)	108 (93.9)	7 (6.1)	0 (0.0)	This study
CPXV-Swe-H1	62 (76.5)	15 (18.5)	4 (5.0)	116 (100)	0 (0.0)	0 (0.0)	This study
CPXV-Swe-H2	61 (77.2)	12 (15.2)	6 (7.6)	108 (98.2)	2 (1.8)	0 (0.0)	This study
CPXV-FIN/T2000	140 (98.6)	2 (1.4)	0 (0.0)	106 (99.1)	0 (0.0)	1 (0.9)	This study
CPXV-BR	0 (0.0)	0 (0.0)	64 (100)	0 (0.0)	0 (0.0)	101 (100)	This study

^a^ATI phenotypes were counted in 50 infected cell sections at random. Values without parenthesis are actual numbers of ATI bodies and the values with the parenthesis are percentage of each phenotype relative to the total number of ATI phenotypes. ATIs have three phenotypes; the V^+^ ATI in which virions are embedded inside the ATI matrix, the V^−^ ATI lacks virions within and on the surface of the ATI matrix, and the V^+/^ ATI in which the virions are not internalized within the inclusion but are encrusted on their surface [32].

### The ***atip*** gene of different geographic CPXV isolates displays distinct XbaI restriction enzyme profiles

**Figure 2:**
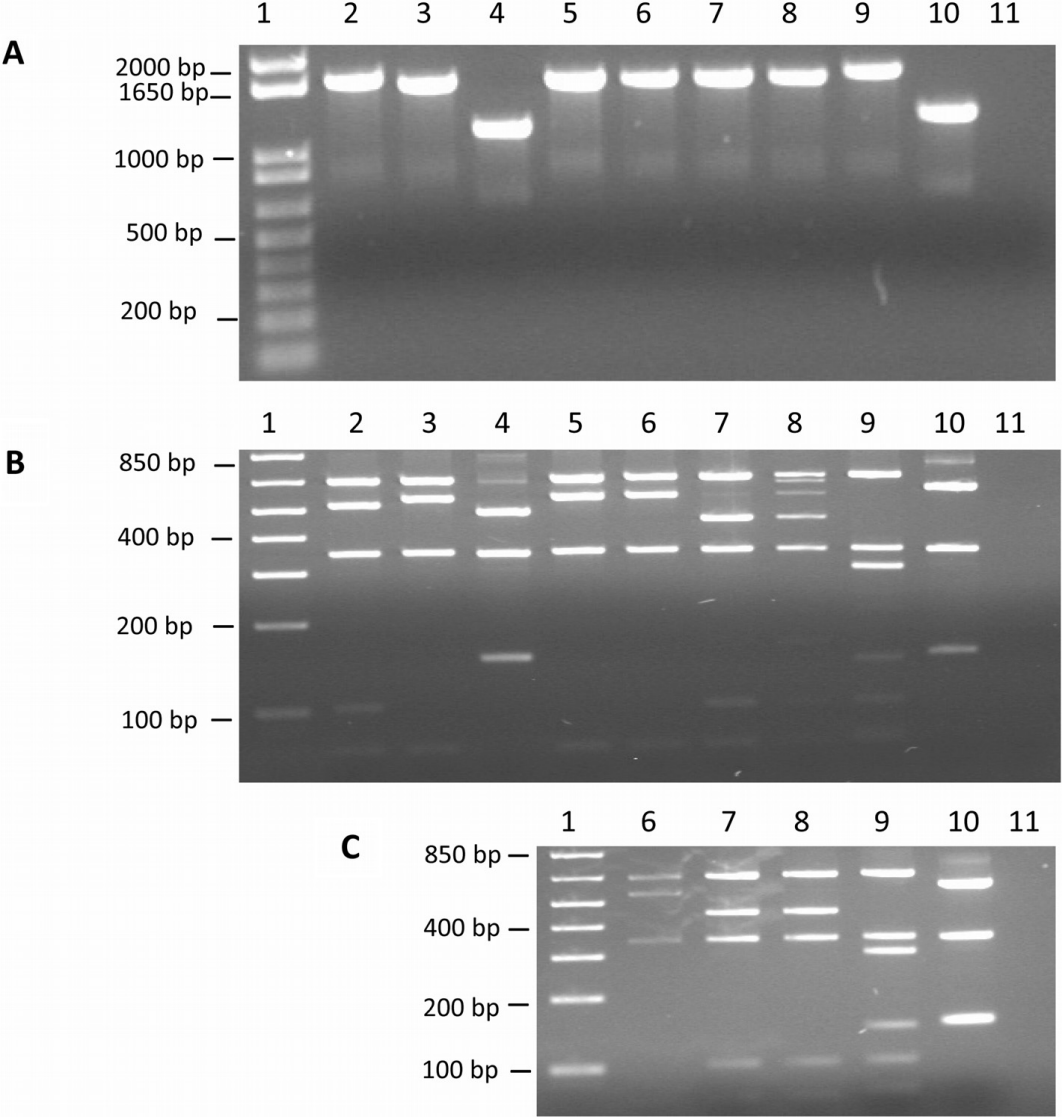
**Amplification and XbaI digestion of *atip *gene fragment. (A)** PCR products of *atip* gene fragment were amplified with ATI-2 primer pairs. **(B)***XbaI* digestion of *atip* gene fragment PCR products for 2 hours. **(C)**. The *XbaI* digestion of *atip* gene fragment PCR products for 4 hours. Lanes (1) 1 kb plus DNA ladder, (2) CPXV-BR, (3) CPXV-No-H1, (4) CPXV-No-H2, (5) CPXV-No-F1, (6) CPXV-No-F2, (7) CPXV-Swe-H1, (8) CPXV-Swe-H2, (9) CPXV-FIN/T2000, (10) ECTV-MOS, (11) dH20.

The XbaI restriction enzyme digestion of PCR products amplified with ATI-2 primer pairs [30,33] has proved to be robust in identifying and differentiating OPVs [30]. To differentiate Fennoscandian CPXVs, we amplified a fragment of the *atip* gene with ATI-2 primer pairs (Figure 2A) and digested the obtained PCR products for 2 hours with XbaI (Figure 2B). Samples showing partial digests after two hours incubation with XbaI were further incubated for another two hours to obtain full digest (Figure 2C). Our results show that an approximate 1659 bp amplicon was generated for CPXV-BR (2) and CPXV-FIN/T2000 (9), 1650 bp product for CPXV-No-H1 (3), CPXV-No-F1 (5), CPXV-No-F2 (6), CPXV-Swe-H1 (7), CPXV-Swe-H2 (8), and approximately 1186 bp and 1279 bp products were amplified for CPXV-No-H2 (4) and ECTV-MOS (10) (Figure 2A). Six groups of XbaI restriction patterns were identified (Figure 2B and C). CPXV-BR belonged to group 1 while CPXV-No-H1, CPXV-No-F1, and CPXV-No-F2 belonged to group 2. Group 3 viruses contained the two Swedish isolates and the Finnish isolate belonged to group 4. Group 5 viruses were made up of CPXV-No-H2 and group 6 contained ECTV-MOS. DNA sequencing of the ATI-2 PCR products in conjunction with *in silico* XbaI digestion of the DNA sequences gave the exact sizes of the amplicons and the XbaI restriction fragments (Table 2). Group 1 (CPXV-BR) produced fragments of 645, 515, 346, 100, and 67 bp); Group 2 (CPXV-No-H1, CPXV-No-F1 and CPXV-No-F2) produced fragments of 645, 543, 346, and 67 bp; Group 3 (CPXV-Swe-H1, CPXV-Swe-H2) generated fragments of 645, 443, 346, 100, and 67 bp) (Table 2). The group 4 viruses (CPXV-FIN/T2000) produced fragments of 645, 345, 299, 144, 100, 72, and 67 bp. The group 5 (CPXV-No-H2) generated fragments of 482, 343, 151, and 151 bp while the group 6 (ECTV-MOS) produced fragments of 575, 343, 151, 151 (Table 2). The restriction pattern of Fennoscandian CPXVs is different from that of the reference strain, CPXV-BR. Thus with the exception of CPXV-No-H2, the restriction pattern of Fennoscandian CPXVs correlates with the geographic origin of the isolates. The CPXV-No-H2 restriction pattern is similar to that of ECTV-MOS. Characterization of CPXV-No-H2 as a naturally occurring recombinant between ECTV and CPXV has been reported elsewhere [33].

**Table 2:** **The *in silico *XbaI restriction enzyme digestion of ATI-2 DNA sequences**

**Virus strain**	**Amplicon size (bp)**	**XbaI digest fragments (bp)**^**b**^	**RFLP profile group**
CPXV-BR	1673	645, 515, 346, 100, 67	1
CPXV-No-H1	1601	645, 543, 346, 67	2
CPXV-No-F1	1601	645, 543, 346, 67	2
CPXV-No-F2	1601	645, 543, 346, 67	2
CPXV-Swe-H1	1601	645, 443, 346, 100, 67	3
CPXV-Swe-H2	1601	645, 443, 346, 100, 67	3
CPXV-FIN/T2000	1672	645, 345, 299, 144, 100, 72, 67	4
CPXV-No-H2	1127	482, 343, 151, 151	5
ECTV-MOS	1220	575, 343, 151,151	6

^b^The ATI-2 PCR products generated with ATI-2 primer pairs as described in Methods were sequenced and the exact sizes of the amplicons were derived from the sequences while the exact sizes of the restriction fragments were obtained by *in silico* digestion of the ATI-2 DNA sequences with XbaI as implemented in Chromas Pro 1.7.5 (http://technelysium.com.au/?page_id=27).

### Fennoscandian CPXVs encode intact ***atip*** and ***p4c*** ORFs

All the Fennoscandian CPXVs (except CPXV-No-H2) produced wild type V^+^ ATI in infected cells and since functionally intact *atip* and *p4c* genes are essential for the formation of V^+^ ATI [23,25], we wanted to find out whether these two genes are intact or disrupted in CPXVs isolated from Fennoscandia. We therefore sequenced the region spanning the *atip* and *p4c* genes as well as their flanking sequences and identified the *atip* and *p4c* ORFs. The Fennoscandian CPXV isolates contained intact *atip* ORFs encoding a polypeptide of 1258 amino acids (aa) in two Norwegian feline isolates, 1262 aa in the two Swedish isolates and 1280 aa in the Finish isolate [HQ680374-HQ680378]. Compared to the reference strain CPXV-BR, the *atip* gene of CPXV isolates from Norway and Sweden contained a 72 bp deletion, but this deletion was absent in the Finnish isolate (Additional file 1). The 72 bp deletion is also present in the *atip* gene of other OPV species including strains of CMLV, MPXV, TATV, VACV and VARV (Additional file 1). The p4c ORFs of the Swedish isolates encoded a polypeptide of 529 aa, CPXV-No-F1 and CPXV-No-F2 contained a polypeptide of 522 aa and 517 aa respectively, and that of the Finnish isolate has a *p4c* coding region corresponding to 518 aa [GenBank accession numbers HQ680374-HQ680378]. The P4c protein is highly conserved among strains of OPVs [24]. Variability in the P4c aa sequence of Fennoscandian CPXVs and other OPVs was present only in the C-terminally located polyaspartate tract (Additional file 2). The number of consecutive aspartic acid residues in the P4c polypeptide of CPXV-No-F1, CPXV-No-F2, CPXV-Swe-H1, and CPXV-FIN/T2000 was 24, 19, 31, and 20 respectively (Additional file 2). In CPXV-Swe-H2 a valine residue was sandwiched between 10 and 20 consecutive aspartic acid residues (Additional file 2). Compared to the Fennoscandian CPXVs, the reference strain CPXV-BR has a disrupted *p4c* ORF due a single nucleotide (Adenine) deletion at position 765 of the alignment (Additional file 3). This deletion resulted in a frame shift mutation that introduced a premature stop codon (TAG) at positions 782 – 784 of the alignment (Additional file 3) and this in turn truncated the CPXV-BR *p4c* gene into CPXV-BR 161 and CPXV-BR 159 ORFs (http://poxvirus.org/gene_detail.asp?gene_id=41865, Additional file 3). A disrupted *p4c* gene is responsible for the V^−^ ATI produced in cells infected with CPXV-BR [23] The presence of full length *atip* and *p4c* genes in CPXVs isolated from Fennoscandia may account for their production of V^+^ ATIs in infected cells.

### Phylogeny, genetic and patristic distances based on the ***atip*** gene

**Figure 3:**
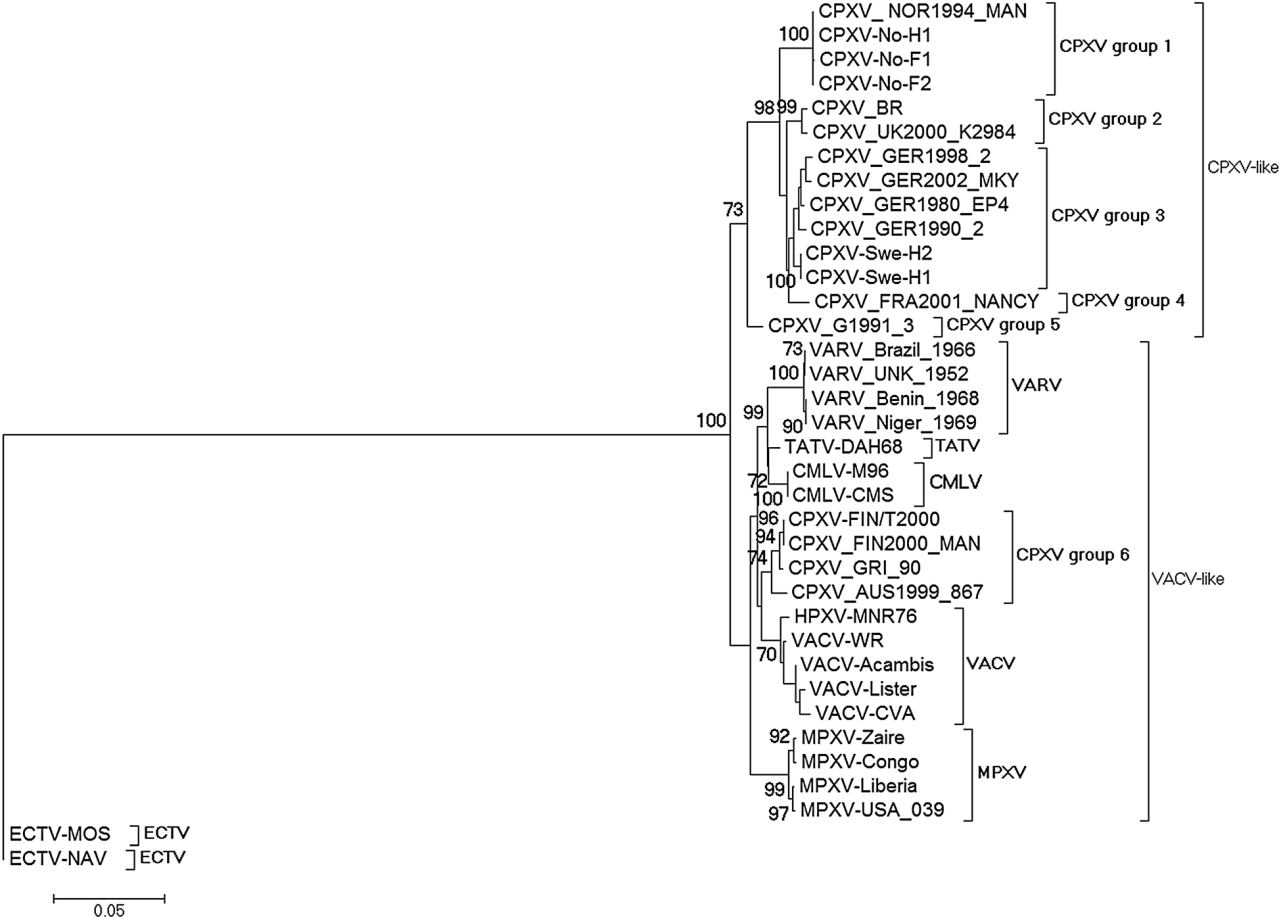
**Maximum Likelihood (ML) phylogenetic tree based on the nucleotide sequence of a complete *atip *ORF.** The ML tree was constructed with MEGA 5.05 as detailed in Methods. Bootstrap values were determined from 1000 replica sampling and only bootstrap values above 70% are shown. The scale indicates substitution per site. Neighbor Joining (NJ) tree constructed with MEGA 5.05 gave identical tree topologies and similar bootstrap support as the ML trees but Bayesian Inference (BI) tree generated with Mr. Bayes 3.1.2 was poorly resolved. CPXVs (14 in total) belonging to clusters (groups) 1 to 5 are CPXV-like because the descended from the same common ancestor as the reference strain CPX-BR. CPXV group 6 are VACV-like because they are closer to VACV than to CPXV-like CPXVs and share a common ancestor with VACV.

To examine the evolutionary relationships between CPXVs isolated from Fennoscandia with other CPXVs/OPVs, the individual alignments of the *atip* and *p4c* nucleotide sequences were subjected to phylogenetic analysis using Maximum - Likelihood (ML), Neighbor – Joining (NJ) and Bayesian – Inference (BI) methods. The *atip* and *p4c* sequences were not concatenated because the Kishino-Hasegawa (KH), Shimodaira-Hasegawa (SH) and Approximately Unbiased (AU) paired tests showed that the phylogenetic signals from the two genes were significantly incongruent (P < 0.05) as to warrant separate analysis. CPXV-No-H2 was excluded from evolutionary analysis of the *atip* gene because a recombination event was detected just downstream the start of the *atip* gene [33]. In addition to tree topology, CPXVs were also separated into different clusters on the basis that the genetic and patristic distances between different clusters or groups were equal or more than the TATV-CMLV threshold. The TATV-CMLV threshold was chosen because it represents the lowest distance between distinct OPV species and thus can serve as the lowest reference distance for grouping OPVs into the same or different species. The ML tree based on the *atip* gene showed that CPXVs were divided into six monophyletic clusters (Figure 3). Group 1 is made up of Norwegian isolates, group 2 contains isolates from the United Kingdom, group 3 included Swedish isolates and some German isolates, group 4 and group 5 were made of one French and one German isolates, while group 6 was made up of CPXV isolates from Finland, Russia and Austria (Figure 3). Both the patristic and genetic distances for each of these groups or clusters were equal to or exceeded the TATV-CMLV threshold (Table 3). The TATV-CMLV patristic distance cutoff as well as the TATV-CMLV genetic distance threshold is 0.013 for the *atip* gene. CPXV groups 1–5 are described as CPXV-like because they are closer to reference CPXV isolate (CPXV-BR) while CPXV group 6 are VACV-like because they are closer to VACV than to any other CPXV including CPXV-BR. Indeed, the patristic and genetic distances between CPXV group 6 (CPXV-FIN/T2000, CPXV_FIN2000_MAN, CPXV_GRI_90, CPXV_AUS1999_867) and VACV (VACV-WR, VACV-Acambis, VACV-lister, VACV-CVA) were 0.026 and 0.022 but the patristic distance between CPXV 6 and any other cluster (CPXV1-5) ranged from 0.038 to 0.061 while the genetic distance varied from 0.032 to 0.049 (Table 3). The NJ tree topology was similar to trees generated by the ML method while the BI trees did not show good resolution like the ML and NJ trees (data not shown). In addition, phylogeny based on the ATIP amino acid sequences yielded tree topologies that were similar to those based on *atip* nucleotide sequences (data not shown). Overall the *atip* gene phylogeny showed that CPXVs resolved into two major monophyletic clades (CPXV-like and VACV-like) and these two major clades were further resolved into six monophyletic clusters or groups. The CPXVs isolated from Fennoscandia were resolved into three of the six monophyletic clusters.

**Table 3:** **The *atip *gene patristic (values without parenthesis) and pairwise genetic distances (values within parenthesis) between distinct clusters of cowpox viruses**

**Clusters**^**c**^							
	**CPXV1**	**CPXV2**	**CPXV3**	**CPXV4**	**CPXV5**	**CPXV6**	**VACV**
CPXV 1							
CPXV 2	0.027 (0.023)						
CPXV 3	0.026 (0.022)	0.017 (0.015)					
CPXV 4	0.028 (0.024)	0.018 (0.016)	0.016 (0.013)				
CPXV 5	0.036 (0.032)	0.033 (0.030)	0.032 (0.025)	0.034 (0.025)			
CPXV 6	0.061 (0.049)	0.058 (0.047)	0.057 (0.040)	0.059 (0.044)	0.038 (0.032)		
VACV	0.067 (0.046)	0.064 (0.042)	0.063 (0.037)	0.065 (0.039)	0.044 (0.029)	0.026 (0.022)	

^c^Patristic distance of the Maximum Likelihood (ML) tree and the genetic distance without reference to a tree were generated as described in the Methods. Distances (patristic and genetic) were averaged across taxa to produce a value for each node. CPXVs belonging to the same cluster have distance values less than TATV-CMLV threshold, while CPXVs belonging to different cluster have distance values equal or more than the TATV-CMLV threshold. TATV-CMLV patristic distance threshold is 0.013 while its genetic distance threshold is also 0.013. CPXV 1 (CPXV_NOR1994_MAN, CPXV-No-H1, CPXV-NoF1, CPXV-No-F2); CPXV 2 (CPXV-BR, CPXV_UK2000_K2984); CPXV 3 (CPXV_GER 1998_2, CPXV_GER2002_MKY, CPXV_GER1980_EP4, CPXV_GER1990_2, CPXV-Swe-H2, CPXV-Swe-H1); CPXV 4 (CPXV_FRA_NANCY); CPXV 5 (CPXV_GER 91–3); CPXV 6 (CPXV-FIN/T2000, CPXV_FIN2000_MAN, CPXV_GRI_90, CPXV_AUS1999_867); VACV (VACV-WR, VACV-Acambis, VACV-lister, VACV-CVA).

### Phylogeny, genetic and patristic distances based on the ***p4c*** gene

**Figure 4:**
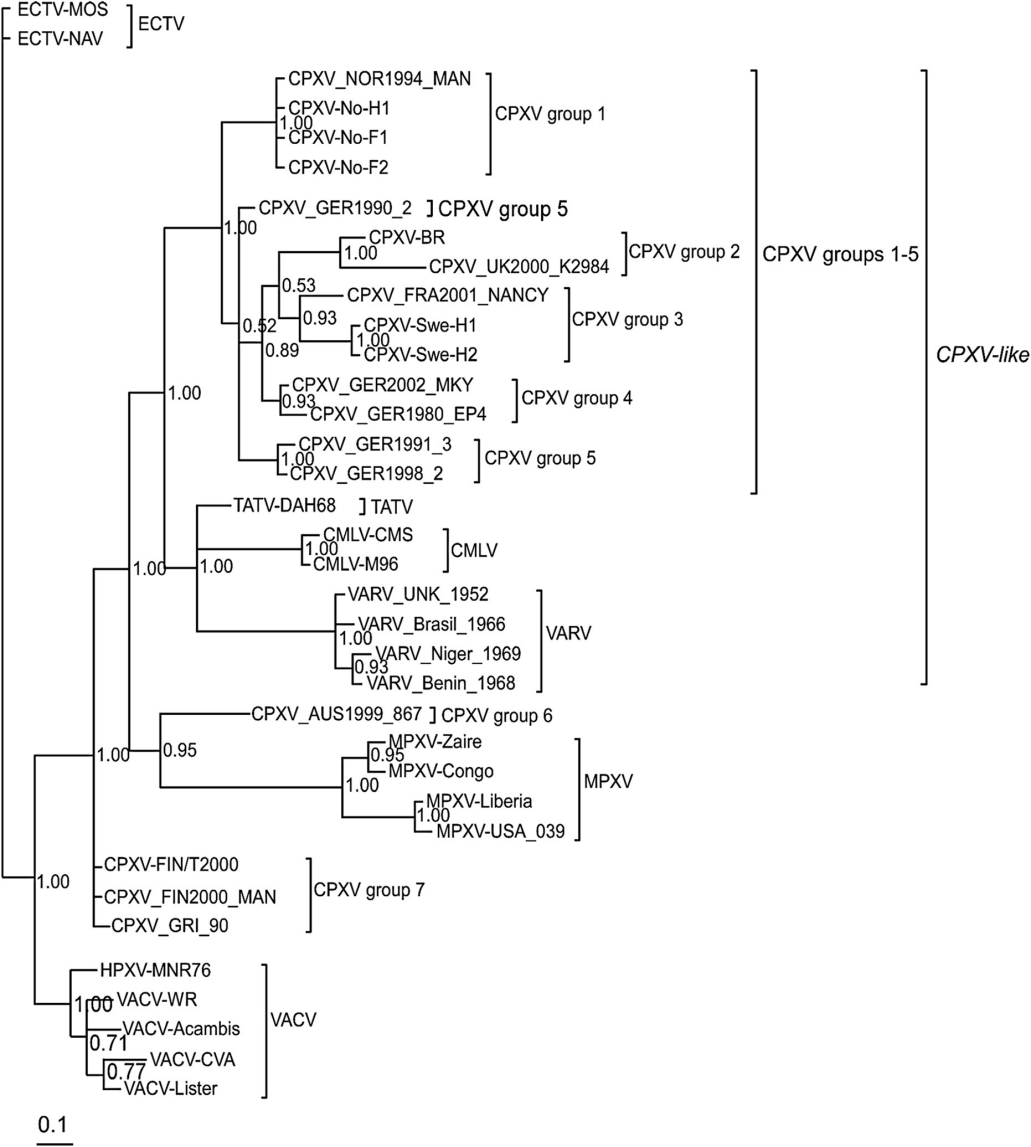
**Bayesian Inference (BI) *p4c *phylogeny with ECTV as outgroup taxa.** The BI tree was constructed with Mr Bayes 3.1.2 as detailed in Methods. Posterior probabilities are shown on the right side of each node. The scale bar represents expected substitutions per site. The topology of phylogenetic trees constructed with Neighbor Joining (NJ) method as implemented in MEGA 5.0.5 was similar to the BI and ML trees. CPXVs (14 in total) belonging to clusters (groups) 1 to 5 are CPXV-like because the descended from the same common ancestor as the reference strain CPX-BR. CPXV groups 6 and 7 (4 viruses in total) are VACV-like because they are closer to VACV than CPXV-like viruses (groups 1 to 5).

**Figure 5:**
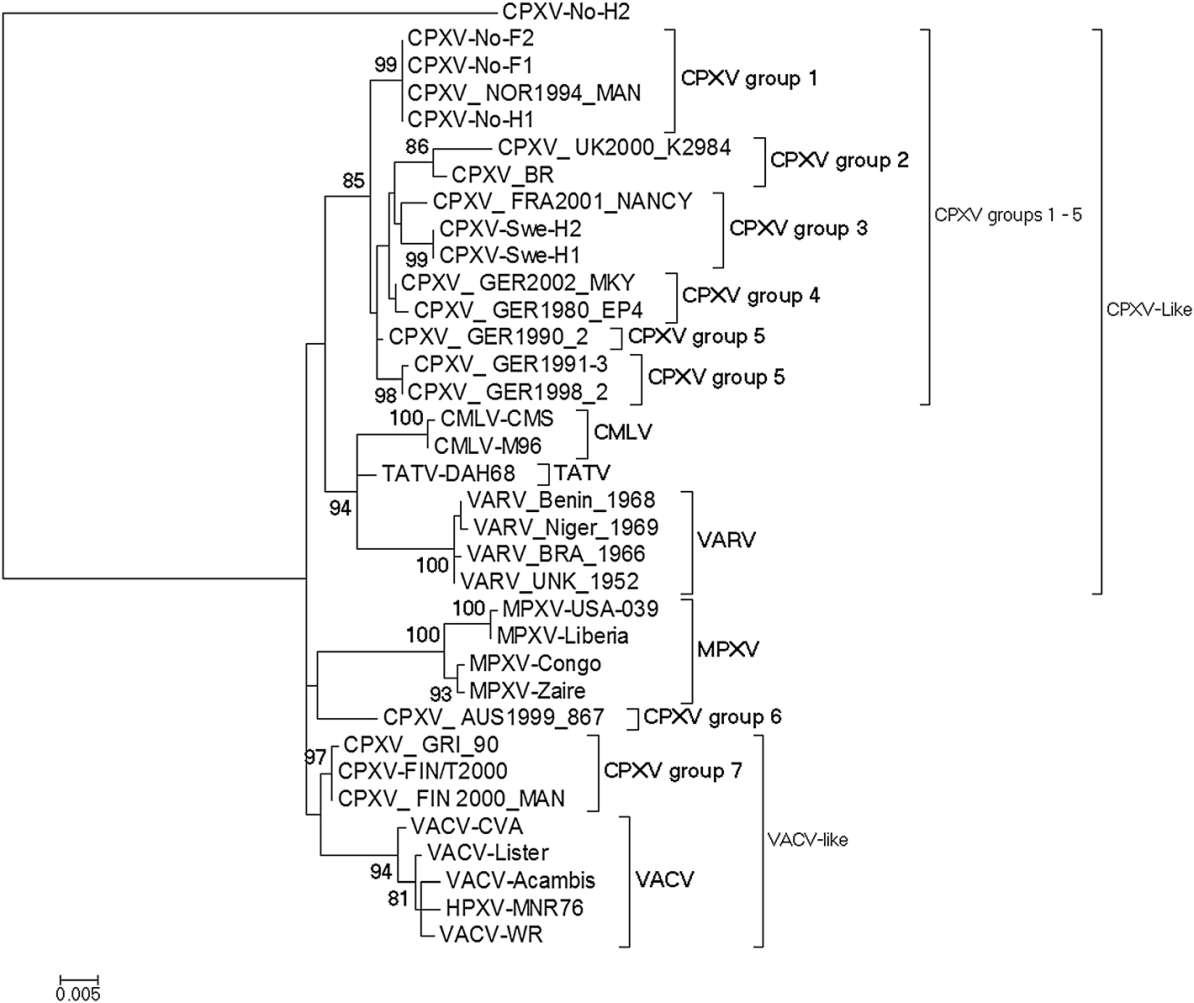
**The *p4c *gene Maximum Likelihood (ML) phylogenetic tree with CPXV-No-H2 as outgroup.** The ML tree was constructed with MEGA 5.05 as outlined in Methods. ECTV sequences were excluded from the analysis. Bootstrap analysis with 1000 replicates was performed and only bootstrap values above 70% are shown. The scale represents substitutions per site. Neighbor Joining (NJ) tree and Bayesian Inference (BI) trees constructed with MEGA 5.05 and Mr Bayes 3.1.2 have tree topologies similar to the ML tree generated with MEGA 5.05. CPXVs (14 in total) belonging to clusters (groups) 1 to 5 are CPXV-like because they descended from the same common ancestor as the reference strain CPX-BR. CPXV groups 6 and 7 (4 viruses in total) are VACV-like because they are closer to VACV than CPXV-like viruses (groups 1 to 5). CPXV group 7 and VACV share a common ancestor.

Taken into cognizance the results of the Kishino-Hasegawa (KH), Shimodaira-Hasegawa (SH) and Approximately Unbiased (AU) paired tests, it was expected that the *p4c* phylogenetic tree topology would be different from that of the *atip* in some nodes and branches. The *p4c* gene phylogenetic tree was constructed with ECTV (Figure 4, Additional file 4) and CPXV-No-H2 (Figure 5) as outgroup taxa as well as an unrooted tree (without assumptions on common decent) in which both ECTV and CPXV-No-H2 were excluded (Additional file 5). The BI tree (Figure 4) and the ML trees (Additional file 4, Figure 5, and Additional file 5) based on the *p4c* gene resolved CPXVs into seven clusters or groups. The patristic and genetic distances between any of the seven clusters were in general equal to or exceeded the TATV-CMLV threshold although there were a few exceptions (Table 4). The TATV-CMLV patristic and genetic distance thresholds were 0.011 and 0.012 respectively for the *p4c* gene (Table 4). The CPXV isolates from Norway and the United Kingdom resolved into CPXV groups 1 and 2 respectively, similar to what was obtained with the tree based on the *atip* gene. However, unlike the *atip* gene phylogeny, CPXV 3 contained the Swedish and French isolates while two and three German isolates belonged to CPXV 4 and CPXV 5 (Figure 4, Additional file 4, Figure 5 and Additional file 5). In addition, the CPXV 6 as obtained with the *atip* based phylogeny was split into groups 6 and 7 in the *p4c* based phylogeny. Group 6 contained a single isolate from Austria while group 7 was made up of isolates from Russia and Finland (Figure 4, Additional file 4, Figure 5 and Additional file 5). The group 7 CPXVs (CPXV-FIN/T2000, CPXV_FIN2000_MAN, and CPXV_GRI_90) was closer to VACV than any other CPXV clusters (Figure 5, Additional file 5). Thus the patristic and genetic distances between CPXV 7 and VACV was 0.013 and 0.014, whereas the patristic and genetic distances between other CPXV clusters (groups 1–6) and VACV had a range of 0.023 – 0.029 and 0.021 – 0.029 (Table 4). Unlike the *p4c* phylogenetic tree rooted with CPXV-No-H2 (Figure 5) and the unrooted tree (Additional file 5), the *p4c* gene phylogeny with ECTV as the outgroup taxa (Figure 4 and Additional file 4) appear not to corroborate the observation that CPXV group 7 is closer to VACV than to any other CPXV cluster. Although the BI (Figure 4) and ML (Additional file 4) phylogenetic trees have similar topology, the BI tree has very strong bootstrap support in almost all the nodes (posterior probability of 1.0), while the ML tree has low clade support (<70%) in some of the nodes. The NJ tree topology for the *p4c* gene was similar to that of the BI and ML trees (data not shown). Also, the tree topologies obtained for the P4c amino acids were similar to what was obtained with the *p4c* nucleotide sequences (data not shown). Overall the phylogeny based on the *p4c* gene as well as the *atip* gene has demonstrated the profound genetic heterogeneity among CPXVs, dividing CPXVs into six or seven distinct clusters. The Fennoscandian CPXVs were found to belong to three of the seven distinct clusters.

**Table 4:** **The *p4c *gene patristic (values without parenthesis) and pairwise genetic distance (values within parenthesis) between clusters of cowpox viruses**

**Clusters**^**d**^								
	**CPXV 1**	**CPXV 2**	**CPXV 3**	**CPXV 4**	**CPXV 5**	**CPXV 6**	**CPXV 7**	**VACV**
CPXV 1								
CPXV 2	0.013							
	(0.013)							
CPXV 3	0.012	0.013						
	(0.012)	(0.014)						
CPXV 4	0.007^*^	0.009^*^	0.008^*^					
	(0.008)^*^	(0.011)^*^	(0.007)^*^					
CPXV 5	0.007^*^	0.011	0.010^*^	0.005^*^				
	(0.007)^*^	(0.014)	(0.009)^*^	(0.006)^*^				
CPXV 6	0.019	0.024	0.023	0.018	0.019			
	(0.020)	(0.024)	(0.020)	(0.019)	(0.019)			
CPXV 7	0.014	0.020	0.019	0.014	0.014	0.014		
	(0.014)	(0.022)	(0.020)	(0.013)	(0.014)	(0.013)		
VACV	0.023	0.029	0.028	0.023	0.024	0.023	0.013	
	(0.021)	(0.029)	(0.029)	(0.023)	(0.024)	(0.025)	(0.014)	

^d^Patristic distance of the Maximum Likelihood (ML) tree and the genetic distance without reference to a tree were generated as described in Methods. Distances (patristic and genetic) were averaged across taxa to produce a value for each node. CPXVs belonging to the same cluster have distance values less than the TATV-CMLV threshold, while CPXVs belonging to different clusters have distance values equal or more than the TATV-CMLV threshold. TATV-CMLV patristic distance threshold is 0.011 while it’s pairwise genetic distance is 0.012. ^*^CPXVs whose distances were less than the TATV-CMLV threshold but were still classified as belonging to different clusters because the distances between one or more individual viruses (as opposed to average distance across taxa) were equal or more than the TATV-CMLV threshold. CPXV 1 (CPXV_NOR1994_MAN, CPXV-No-H1, CPXV-No-FI, CPXV-No-F2); CPXV 2 (CPXV-BR, CPXV_UK2000_K2984); CPXV 3 (CPXV_FRA2001_NANCY, CPXV-Swe-H1, CPXV-Swe-H2), CPXV 4 (CPXV_GER2002_MKY, CPXV_GER1980_EP4); CPXV 5 (CPXV_GER1991_3, CPXV GER1998_2); CPXV 6 (CPXV_AUS1999_867); CPXV 7 (CPXV-FIN/T2000, CPXV_FIN2000_MAN, CPXV_GRI_90); VACV (VACV-WR, VACV-Lister, VACV-Acambis, VACV-CVA, HPXV-MNR76).

## Discussion

The objective of this study was to characterize CPXVs isolated from Fennoscandia and compare their biological and genetic characteristics to that of CPXVs isolated from other geographic regions, as well as to other OPV species. The Fennoscandian CPXVs were characterized on the basis of ATI phenotype, RFLP of *atip* gene fragment amplicon, sequence and phylogenetic analysis based on full length *atip* and *p4c* genes. We have demonstrated that CPXVs isolated from Fennoscandia produced wild type V^+^ ATI (except for CPXV-No-H2) and encode full length *atip* and *p4c* genes. The V^+^ ATI was produced in both Vero and A549 cells, suggesting that it is a strain specific trait. Functionally intact *atip* and *p4c* genes have been shown to be essential [23] but not sufficient [24,33] for the formation of V^+^ ATI. In addition to functionally intact *atip* and *p4c* genes, it has been demonstrated that VACV Copenhagen *A27L* homologue is required for the formation of V^+^ ATI [25]. The production of V^+^ ATI in cells infected with Fennoscandian CPXVs may be due to the presence of functionally intact *atip*, *p4c* and *A27L* sequences. Although these three genes have been shown to be essential for the formation of V^+^ ATI, it cannot be excluded that other genes are involved in the formation of this phenotype. We are currently investigating whether the three genes (*atip, p4c*, *A27L* homologue) are sufficient for the production of wild type V^+^ ATI or if additional genes are required. The occlusion of virions within ATI may facilitate host to host transmission by protecting the virions from the harsh environment during transmission between hosts [26]. Thus, Fennoscandian CPXVs with the exception of CPXV-No-H2 may have evolved the V^+^ATI phenotype to increase virus survival capability outside the host and aid host-to-host transmission under extreme climatic conditions in Fennoscandia, especially the seasonal freeze-thaw cycles.

The RFLP profiles generated by digesting PCR products amplified with ATI-2 primer pairs have enabled correct species assignment of 73 OPV isolates already known to belong to different OPV species [34]. Our application of this method to CPXVs isolated in Fennoscandia yielded RFLP profiles that correlate with the geographic region of the isolates. Although the RFLP profiles 2–4 were unique to Fennoscandian CPXVs, they were more or less related to published profiles of other CPXVs [34]. However the RFLP profile 5 for CPXV-No-H2 was similar to that of ECTV and was completely different from any known CPXV profile. This observation was the first clue of a suspected recombination event. We have reported elsewhere that CPXV-No-H2 is a novel recombinant between CPXV and ECTV [33]. Thus, in addition to robust differentiation and classification of OPV strains, RFLP profiles generated by digesting ATI-2 primer amplicons can serve as a first indicator of atypical or recombinant CPXV.

The phylogenetic tree topology in tandem with genetic and patristic distances has been used for robust molecular taxonomy of OPVs [20]. We employed the same method in classifying Fennoscandian CPXVs and other CPXVs/OPVs, with the modification that we chose to use the TATV-CMLV threshold rather than VARV-TATV that was reported by others [20]. We chose the TATV-CMLV threshold because TATV is closer to CMLV than to VARV [35], thus the distances between TATV and CMLV represents the lowest distance between distinct OPV species, and CPXVs whose genetic/patristic distances equals or exceeds the TATV-CMLV threshold should not be grouped as the same species. The *atip* gene phylogeny showed that OPVs were divided into two major monophyletic clades that were further subdivided into six clusters. The CPXV-like clade was exclusively made of five different CPXV clusters (CPXV 1–5) that did not contain any other OPV species, while the VACV-like clade contained VACV-like CPXVs (CPXV 6) in addition to MPXV, VACV, TATV and CMLV. These results are in agreement with a recent phylogenetic analysis based on ten concatenated conserved genes [27]. However, it has to be noted that the bootstrap support for the VACV-like clade is low. One possible explanation for the low VACV-clade support is that vaccine or laboratory strains of VACV were used for the construction of the phylogenetic tree. These vaccine/laboratory strains usually have high number of passages in cell cultures or laboratory animal, and these passages may have introduced mutations into the genome of these strains. Presumably, these mutations might have interfered with the phylogenetic signals. To test this hypothesis; we reconstructed the *atip* gene phylogeny using sequences of naturally isolated VACV strains. The most improved VACV clade support was obtained when only HSPV-MNR76 and VACV-3737 sequences were used. VACV clade support was improved from less than 50% (Figure 3) to 61% (Additional file 6) while the CPXV-clade was improved from 73% (Figure 3) to 82% (Additional file 6). These results suggest that mutations introduced in the *atip* gene of vaccine/laboratory strains of VACV following passages in cell culture or laboratory animals may in part account for the low VACV clade support. Alternatively, low clade support may indicate that members of the clade are not phylogenetically related. However this is unlikely in this case as phylogenetic analysis based on multiple genes as well as the entire conserved central region of OPV genome have yielded tree topology consistent with the *atip* gene phylogeny reported in this study with the exception that bootstrap support for the VACV-like clade was 95% or more [27]. It has been estimated that CPXV-like and VACV-like clades of OPVs diverged from the common ancestor some ten thousand years ago (TYA) [27], a period that corresponds to the last ice age over Fennoscandia. It has been speculated that the wide abundance of various rodents (natural host to CPXVs and other OPVs) may have fuelled OPV divergent evolution. However, full genome OPV phylogeny grouped CPXVs classified as CPXV-like as sister to CMLV, TATV and VARV. This is in disagreement with the *atip* gene phylogeny reported in this paper and also with phylogeny based on concatenated conserved genes published elsewhere [27]. The reason(s) for this discrepancy is unclear but it may be that phylogeny based on whole genome provides better resolved trees than those obtained with single genes or concatenated multiple genes. Again, while the *atip* gene phylogeny reported in this study resolved CPXV_NOR_1994_MAN into the same species cluster as other CPXVs isolated from Norway (CPXV group 1) and distinct from isolates from the United Kingdom (CPXV group 2), the genome based phylogenetic tree grouped CPXV_NOR_1994_MAN into the same cluster as isolates from the United Kingdom [20]. There are two likely reasons for this. First, only one isolate from Norway and no isolate from Sweden were used in the whole genome phylogenetic tree reconstruction. This might have under-represented phylogenetic signals for CPXVs isolated from Norway. Secondly, the higher TATV-VARV threshold was used in the whole genome study as opposed to the lower TATV-CMLV threshold that was used in this study. Indeed if the lower TATV-CMLV threshold was used in the whole genome study, CPXV_NOR_1994_MAN would be distinct from isolates from the United Kingdom. Thus, it can be concluded that CPXV_NOR_1994_MAN clustered with other CPXV isolates from Norway and is distinct from CPXVs isolated in the United Kingdom.

The phylogeny based on the *p4c* gene showed that CPXVs classified as CPXV-like (CPXV group 1–5) formed a major OPV monophyletic clade that included CMLV, TATV and VARV. Thus, as opposed to the *atip* gene phylogram, the *p4c* tree shows that CPXVs classified as CPXV-like (CPXV group 1–5) were sister to TATV, CMLV and VARV. This is in accordance with the results of whole genome phylogeny [20], but in contrast to the phylogram obtained from concatenating conserved genes located at the central region of the genome [27]. In addition, the *p4c* genetic and patristic distance measures showed that CPXV group 7 was closer to VACV than to any other OPV. Surprisingly, the *p4c* gene phylogeny with ECTV as outgroup taxa (Figure 4, Additional file 4) does not corroborate the findings obtained from patristic and genetic distance measures. We suspected that the reason for this is that the ECTV sequence is not an uncontroversial outgroup taxa as it is too close to VACV. We tested this hypothesis by reconstructing an unrooted *p4c* gene phylogenetic tree and our result confirmed that the ECTV *p4c* sequence was very close to the VACV (data not shown). When ECTV was excluded and an unrooted tree was reconstructed, it clearly showed that CPXV group 7 formed the same monophyletic clade as VACV (Additional file 5). Previously, we have shown that the *p4c* gene of CPXV-No-H2 is diverged from the homologues in all OPV species and probably represent an ancestral sequence [33]. A reconstruction of the *p4c* gene phylogeny with CPXV-No-H2 sequence as out group taxa demonstrated that CPXV group 7 formed the same clade with VACV (Figure 5). This is in agreement with the whole genome phylogeny [20]. The evidence that CPXV-No-H2 has ECTV *atip* gene while its *p4c* gene was shown to be distant from other CPXVs [33] (Figure 5) may raise the suspicion that it is not a CPXV strain. In addition to clinical history we have shown that CPXV-No-H2 is a CPXV strain based (i) presence of two copies of *cytokine response modifier B* (*crmB*) gene [18], (ii) sequence and phylogenetic analysis based on multiple genes including *crmB*, *Chinese hamster ovary host range* (*CHOhr*) gene, and the *haemagglutinin* (*HA*) gene [18,33]. Our previous *HA* phylogenetic tree construction did not include all the CPXVs used in this study. Therefore we reconstructed the *HA* phylogenetic tree including all the CPXVs used in this study as well as representatives of both “Old World” and “North America” OPV species. Our result clearly showed that CPXV-No-H2 has phylogenetic affinity with other CPXVs belonging to CPXV-like clade and it is phylogenetically distinct from other “Old World” and “North American” species of OPVs (Additional file 7). Unlike the *p4c* BI phylogenetic tree which has strong posterior probability of 1.0 (100%) in most nodes including the node that grouped CPXV 1–5 with CMLV, TATV and VARV, the *p4c* ML tree has low bootstrap support in some of nodes. The low clade support in some nodes in the *p4c* gene ML tree may be due to under-estimation of clade support by the ML algorithm.

The conflicting phylogenetic signals between the *atip* and *p4c* genes as shown by both the paired tests and some of the branching and nodes in the tree topologies were rather surprising since both genes are located in the same central part of the genome (the *p4c* gene is just upstream of the *atip*) and are required for the same function, that is, the formation of ATI phenotypes [25]. The conflicting phylogenetic signals between *atip* and *p4c* genes may indicate that either or both genes are involved in functions other than the formation of ATI, and thus may have different rates of evolution. In spite of this conflict in phylogenetic signals between the *atip* and *p4c*, both phylograms have demonstrated that (i) CPXVs are genetically heterogeneous and can be subdivided into six or seven species clusters, (ii) CPXVs isolated from Fennoscandia belong to three of the distinct clusters and that each of the clusters contain isolates from one specific geographic region or country, (iii) CPXV_NOR_1994_MAN belonged to the same cluster as other Norwegian isolates and was distinct from isolates from the United Kingdom, (iv) VACV-like CPXVs (CPXV_GRI_90, CPXV-FIN/T2000, CPXV_FIN2000_MAN, CPXV_AUS1999_867) are closer to VACV than any of the other 14 CPXV isolates (CPXV group 1–5), (v) TATV, CMLV and VARV belong to the same monophyletic cluster, and TATV is closer to CMLV than to VARV, (vi) HSPV is grouped together with VACV isolates, and (vii) MPXVs were resolved into Congo basin and West Africa sub-clusters. Other investigators have demonstrated similar results with respect to OPV phylogeny and evolution [17,35-37].

The fact that CPXV isolates from Norway and Sweden were classified as CPXV-like while isolates from Finland in conjunction with the isolate from Russia were VACV-like may be an indication that CPXV-like and VACV-like CPXVs have two distinct evolutionary histories in geographically or otherwise separated rodent lineages that re-colonized Fennoscandia after the post glacial retraction 12 – 8 TYA. A recent retrospective study on OPV molecular evolution showed that CPXV-like viruses may have separated from the common ancestor approximately 10 TYA and commenced individual evolution. This event may have triggered the divergent evolution of OPVs that lead to the emergence of VACV-like CPXVs and other OPV species [27]. Intriguingly, the estimated 10 TYA age for OPV divergent evolution corresponds well to the time of post glacial retraction (12 – 8 TYA), thus supporting the post glacial retraction hypothesis for OPV evolution. Moreover, post-glacial re-colonization hypothesis has been used to explain the distribution of different Puumala Hantavirus (PUU) genotypes in Norway and Sweden [38,39], and it is interesting that both PUU and CPXV have bank voles as reservoir species [38-40]. Detailed studies including a large number of CPXVs isolated from different geographic regions in Fennoscandia as well as their genome sequences will be required in order to determine whether or not (or to what degree) post-glacial re-colonization could explain the genetic diversity among CPXVs.

## Conclusions

CPXVs isolated from Fennoscandia encode full length *atip* and *p4c* genes and produced wild type V^+^ ATI except for CPXV-No-H2. CPXVs were genetically heterogeneous and were resolved into six or seven distinct clusters. The Fennoscandian CPXV belonged to three of the distinct clusters and isolates from the same country or geographic region belonged to the same cluster. Our results show that VACV-like CPXVs and CPXV-like CPXVs may have distinct evolutionary histories, presumably in rodent host species. These results are of relevance to the evolution and classification of CPXVs in particular and OPVs in general.

## Methods

### Cells and viruses

Vero (ATCC CCL-81) and A549 (ATCC CCL-185) were obtained from the American Type Culture Collection (ATCC) (Rockville, MD, USA). Both cell lines were grown under conditions suggested by ATCC. The origin of Fennoscandian CPXV isolates used in this study has been described elsewhere [41-43]. The Swedish CPXV isolates designated Swe-H1 and Swe-H2 were isolated from human clinical cases in the district of Skåne in 1990 [43]. The Norwegian isolates No-H1 and No-FI were isolated in 1994 from a human and a cat in the district of Bergen [42]. The Norwegian isolate, No-F2, was isolated from a cat from the same Bergen district in 1999 [18], and another Norwegian human isolate No-H2 was isolated in 2001 from a male teenager in Nordland, Norway [33]. The Finnish isolate, FIN/T2000, was isolated from a four year old girl in 2000 [41]. The reference strain CPXV Brighton Red (CPXV-BR) was included for comparison/control and ECTV strain Moscow (ECTV-MOS) was included for comparison of RFLP profiles. ECTV-MOS was purchased from ATCC. All isolates except No-F1 and No-F2 have been passaged in cell cultures prior to arrival in our laboratory. The isolates have been passaged once or twice prior to arrival in our laboratory. Stocks of virus isolates were prepared from infected Vero cells after three rounds of plaque purification/amplification and titer of stocks were determined by plaque assay [44].

### Transmission electron microscopy

Confluent monolayers of Vero and A549 cells in six well tissue culture plates (NUNC, Sweden) were infected with Fennoscandian CPXVs and CPXV-BR at a multiplicity of infection (m.o.i.) of 5 plaque forming units (pfu) per cell. Viruses were adsorbed to monolayers for one hour at 4°C. Infected cells were washed twice with phosphate buffered saline (PBS) and incubated in a medium containing 2.5% fetal bovine serum (FBS) at 37°C in a 5% CO_2_ atmosphere. At 36 hours post infection (hpi), infected cells were washed with fresh medium and fixed in MacDowell’s solution, pH7.4, for one hour at room temperature. Fixed cells were processed for transmission electron microscopy as described elsewhere [23]. Quantification of ATI phenotypes by electron microscopy in sections of infected cells was as we previously described [24]. Collection of images from thin sections was done in JEOL electron microscopy operating at an accelerating voltage of 100 K.

### PCR amplification

Viral DNA was extracted from the lowest cell culture passage of viral stocks using DNA Minikit (QIAGEN GmbH Strasse 1, Hilden, Germany). Eight primer pairs at different molar concentrations were used to generate amplicons spanning the entire *atip* and *p4c* ORF as well as their flanking sequences. All the eight primer pairs; ATIN-1, ATIN-3, ATIN-4 [33], ATI-2 [30], ATI-5, p4c-1, p4c-2, p4c-3 [24] have been published previously. The PCR reaction for each gene fragment was performed with 2 ng DNA in a total volume of 50 μl PCR mix. The PCR mixes contained appropriate concentration of primer pairs [33], 200 μM of each dNTP, 1 x reaction buffer (10 mM Tris–HCl, pH 8.3, 50 mM KCL, 1.5 mM MgCl_2_) and 1.25U Amplitaq DNA polymerase (Applied Biosystems, Foster City, USA). The PCR cycling conditions for all the primer pairs have been described elsewhere [24,33]. The PCR products were resolved in 1% Seakem agarose gels (Cambrex Bioscience, Rockland, ME, USA) in 1 x TAE buffer. Pictures of ethidium bromide stained DNA fragments were photographed with Gel Doc 2000 (Biorad).

### Restriction enzyme digestion of ATI-2 PCR product

The ATI-2 gene fragment of Fennoscandian CPXV, CPXV-BR, and ECTV-MOS was amplified from virus DNA as previously described [30,33]. The ATI-2 PCR product was digested with 20 units of XbaI and incubated at 37°C for 2 hours. Amplicons that were not fully digested within the two hour period were subjected to additional two hours of digestion. The restriction digests were separated using 2.0% Metaphore agarose (Medprobe) gels. The RFLP profiles were photographed with Gel Doc 2000 (Biorad).

### DNA sequencing

The PCR products of the eight overlapping fragments for each Fennoscandian CPXVs were purified using GFX PCR DNA and Gel Band Purification Kit (GE Health, Uppsala, Sweden), following the manufacturers instruction. Cycle sequencing reactions were performed using Big Dye 3.1 Sequencing Kit (Applied Biosystems, Foster City, CA, USA). Each purified DNA fragment was sequenced in both orientations and at least two independent DNA cycling reactions were performed for each fragment. The cycle sequencing extension products were electrophoresed using ABI Prism™ 377 DNA automatic sequencer (Applied Biosystems).

### Contig assembly, sequence analysis and recombination detection

Raw sequence data was edited in TREV 1.9 (http://staden.sourceforge.net). Forward and reverse DNA sequences for fragments 1–8 for each of the Fennoscandian CPXVs were assembled into a single contig using Chromas Pro 1.5 software (http://technelysium.com.au/?page_id=27). The *atip* and *p4c* ORFs were identified from respective contigs using ORF finder (http://www.ncbi.nlm.nih.gov/gorf/gorf.html). The *atip* and *p4c* sequences (nucleotide and amino acids) of Fennoscandian CPXV isolates and those of other OPVs (including 12 other CPXVs) (Table 5) were retrieved from the GenBank and aligned with ClustalW version 1.8 [45]. Positions with gaps were removed from the *atip* and *p4c* alignments (nucleotide and amino-acids). The DNA and amino acid identity matrices were calculated from aligned sequences using Bioedit version 7.0.5.3 (http://www.mbio.ncsu.edu/BioEdit/page2.html). For recombination detection, the approximately 6200 nt region containing the *atip* and *p4c* ORFs of Fennoscandian CPXVs, other CPXVs and ECTV-MOS were aligned with ClustalW 1.8 [45], and subjected to recombination analysis. Recombination detection was carried out with SimPlot version 3.5.1 [46] and Recombination detection program 3 (RPD3) [47]. The sequences reported in this paper were deposited under GenBank accession numbers [HQ680372, HQ680374-HQ680378].

**Table 5:** The orthopoxvirus (OPV) strains used in the study

**Virus species**	**Species abbreviation**	**Strain**	**ATI phenotype**^**e**^	**GenBank accession number**
Camelpox virus	CMPV	CMS	V^0^	AY009089
		M-96	V^0^	NC_003391
Cowpox virus	CPXV	No-H1	V^+^	This study
		No-F1	V^+^	This study
		No-F2	V^+^	This study
		Swe-H1	V^+^	This study
		Swe-H2	V^+^	This study
		FIN/T2000	V^+^	This study
		No-H2^#^	V^+/^	HQ680373
		NOR1994_MAN^*^	V^+^	HQ420899
		FIN2000_MAN^*^	V^+^	HQ420893
		Brighton Red	V^−^	NC_003663
		GER 91-3	V^+^	DQ437593
		GRI-90	V^+^	X94355
		GER1990_2	V^+^	HQ420896
		FRA2001_NANCY	V^+^	HQ420894
		UK2000_K2984	V^+^	HQ420900
		AUS1999_867	V^+^	HQ407377
		GER1990_EP4	V^+^	HQ420895
		GER1998_2	V^+^	HQ420897
		GER2002_MKY	V^+^	HQ420898
Ectromelia virus	ECTV	Moscow	V^−^	NC_004105
		Naval	V^−^	-
Monkeypox virus	MPXV	Zaire	V^0^	NC_03310
		Congo	V^0^	DQ011154
		USA_039	V^0^	DQ011157
		Liberia	V^0^	DQ011156
Taterapox virus	TATV	DAH68	V^0^	NC_008291
Horsepox virus	HSPV	MNR76	V^0^	DQ792504
Vaccinia virus	VACV	CVA	V^0^	AM501482
		Lister	V^0^	AY678276
		Acambis	V^0^	AY313847
		Western Reserve	V^0^	NC_006998
Variola virus	VARV	Niger_1969	V^0^	DQ441434
		Benin_1968	V^0^	DQ441416
		Brazil_1966	V^0^	DQ441419
		UNK_1952	V^0^	DQ441447

^e^The ATI phenotypes of Fennoscandian CPXVs, CPXV-BR, ECTV-MOS were determined by electon microscopy as outlined in Methods while that of other OPVs were predicted based on the presence of full length *atip* and *p4c* genes. Strains that do not produce ATI were labelled V^0^. The V^+^ has virions embedded with the ATI matrix while the V^−^ has no virions within or on the surface of the ATI matrix. The V^+/^ ATI have virions encrusted on the surface but not internalized within the matrix. ^#^The V^+/^ ATI of CPXV-No-H2 was demonstrated in our previous report [33]. ^*^CPXV_NOR1994_MAN and CPXV_FIN2000_MAN are clones of CPXV-No-H1 and CPXV-FIN/T2000 whose genomes are fully sequenced [20]. ECTV-NAV has not been assigned a GenBank accession number but its genome sequence is available at http://www.poxvirus.org.

### Phylogenetic analysis, patristic and genetic distance matrices

The best fit nucleotide substitution model was selected based on JmodelTest [48] while the best fit model for protein evolution was selected using ProTest [49]. Kishino-Hasegawa (KH), Shimodaira and Hasegawa (SH), and Approximately Ubiased (AU) paired tests implemented in Treefinder version march 2011 (http://www.treefinder.de/) [50] were used to examine the possibility of concatenating the *atip* and *p4c* aligned sequences for phylogenetic analysis. A significant p-value (P < 0.05) rejects the null hypothesis (all the tree topologies are good explanations of the data) and precludes combining the *atip* and *p4c* molecular datasets. Phylogenetic trees were constructed for *atip a*nd *p4c* alignments using ML and NJ methods as implemented in MEGA version 5.05 [51], BI as implemented in MrBayes version 3.1.2 [52]. ECTV-MOS and ECTV strain Naval (ECTV-NAV) were included as outgroup taxa in all phylogenetic analysis. CPXV-No-H2 *p4c* gene was included as an outgroup for the *p4c* phylogeny. Bayesian analysis was conducted with the following settings: nst = 6, rates = invgamma, mcmc (2 simultaenous runs), samplefreq = 1000, nchains = 4 (3 hot, 1 cold), 10 million generations, and burnin = 2500. A 50% majority rule consensus tree was generated and posterior probabilities of 95% or more was considered statistically significant for clade support [53]. ML gene trees were constructed using GTR + G substitution model while the Maximum Composite Likelihood model was used for the construction of NJ gene trees [51]. The robustness of ML and NJ trees was evaluated by bootstrap analysis of 1000 replicates. The pairwise genetic distance without reference to a tree was generated for the *atip* and *p4c* genes using the Maximum Composite Likelihood method [51] while the patristic distance (tree branch lengths between taxa) between the isolates was extracted from the ML and BI trees using the program Patristic version 1.0 (http://www.bioinformatics.org/patristic/) [54]. For both genetic and patristic distance methods, the distances were averaged across taxa to produce a value for each node. Since TATV and CMLV represent the two closest but distinct OPV species, the distance (genetic and patristic) between these two species represents a threshold value. In addition to tree topology, CPXVs were further separated into different groups if their genetic/patristic distance is equal or exceeds the TATV-CMLV threshold.

## Competing interests

The authors declare that they have no competing interests.

## Authors’ contributions

MIO designed the study, carried out the experiments and wrote the draft of the manuscript. ASO participated in the design of the study, interpreted data and revised the manuscript. ØN contributed to the conception and design of the study, interpreted the data and revised the manuscript. UM interpreted the data, revised the manuscript and participated in the design of the study. MT contributed to the study design, interpreted data and revised the manuscript. TB interpreted the data and revised the manuscript. TT conceived the study, contributed to its design, interpreted data and revised the manuscript. All authors read and approved the final manuscript.

## Supplementary Material

Additional file 1 **Multiple sequence alignment of the DNA sequence of *atip *gene.** Only regions of the alignment depicting the 72 bp deletion in some isolates (compared to that of CPXV-BR) are shown. Click here for file

Additional file 2 **Multiple sequence alignment of the amino acid sequence of the P4c protein.** Only the region encompassing the C-terminal polyaspartate tract of the P4c protein of some strains of OPV is depicted. Click here for file

Additional file 3 **Multiple sequence alignment of the DNA sequence of the *p4c *gene.** Only the region showing the polymorphism that truncated the *p4c* gene of CPXV-BR is shown. Compared to the Fennoscandian CPXVs, the reference strain CPXV-BR has a single nucleotide deletion at position 765 which resulted in a frame shift mutation that introduced a stop codon at position 782–784. This terminated the first open reading frame (CPXV 161). The second open reading frame (CPXV 159) starts with the initiation codon at positions 1003–1005 of the alignment. Click here for file

Additional file 4 **Maximum Likelihood (ML) *p4c *gene phylogeny with ECTV as outgroup taxa.** The ML tree was constructed with MEGA 5.0 as outlined in Methods. Bootstrap analysis with 1000 replicates was performed and only bootstrap values above 70% are shown. The scale represents substitutions per site. Neighbor Joining (NJ) tree and Bayesian Inference (BI) tree constructed with MEGA 5.0 and Mr Bayes 3.1.2 have tree topologies similar to the ML tree generated with MEGA 5.0. CPXVs (14 in total) belonging to clusters (groups) 1 to 5 are CPXV-like because the descended from the same common ancestor as the reference strain CPX-BR. CPXV groups 6 and 7 (4 viruses in total) are VACV-like because they are closer to VACV than CPXV-like viruses (groups 1 to 5). Click here for file

Additional file 5 **Unrooted *p4c *gene phylogenetic tree constructed with Maximum Likelihood method.** The ML tree was constructed with MEGA 5.05 as detailed in Methods. ECTV and CPXV-No-H2 sequences were excluded from the analysis. Bootstrap analysis with 1000 replicates was performed and only bootstrap values above 60% are shown. The scale represents substitutions per site. Neighbor Joining (NJ) tree and Bayesian Inference (BI) trees constructed with MEGA 5.05 and Mr Bayes 3.1.2 have tree topologies similar to the ML tree generated with MEGA 5.05. CPXVs (14 in total) belonging to clusters (groups) 1 to 5 are CPXV-like because they are grouped in the same clade with the reference strain CPXV-BR. CPXV groups 6 and 7 (4 viruses in total) are VACV-like because they are closer to VACV than CPXV-like viruses (groups 1 to 5). CPXV group 7 and VACV are grouped in the same clade. Click here for file

Additional file 6 **The *atip *gene phylogenetic tree constructed with Maximum Likelihood in which vaccine and laboratory strains of vaccinia virus (VACV) were excluded.** Laboratory and vaccine strains (as used in Figure 3) were excluded. Naturally isolated VACVs; HSPV-MNR76 and VACV-3737 (DQ377945) were the only VACV strains included for the construction of the phylogenetic tree. The ML tree was constructed with MEGA 5.05 as described in Methods. Bootstrap values were determined from 1000 replica sampling and only bootstrap values above 60% are shown. The scale indicates substitution per site. Neighbor Joining (NJ) tree constructed with MEGA 5.05 gave similar tree topology as the ML tree but with higher bootstrap support. The Bayesian Inference (BI) tree generated with Mr. Bayes 3.1.2 was poorly resolved. CPXVs (14 in total) belonging to clusters (groups) 1 to 5 are CPXV-like because the descended from the same common ancestor as the reference strain CPX-BR. CPXV group 6 are VACV-like because they are closer to VACV than to CPXV-like CPXVs and share a common ancestor with VACV. Click here for file

Additional file 7 **Neighbor Joining (NJ) tree based on the nucleotide sequences of complete *haemaggluttinin (HA) *ORFs.** The NJ tree was constructed from aligned sequences using MEGA 5.05. Bootstrap values were determined from 1000 replica sampling and only bootstrap values above 60% are shown. The bar indicates the divergence scale. The topology of the Maximum Likelihood (ML) tree constructed with MEGA 5.05 was similar to the NJ tree but with a lower bootstrap support in some of the nodes. Click here for file
