# Supplementary figures and images for: Molecular characterization and phylogenetics of Fennoscandian cowpox virus isolates based on the p4c and atip genes
Source: Virol J. 2014 Jun 27;11:119. doi: 10.1186/1743-422X-11-119 (PMC4112975; doi:10.1186/1743-422X-11-119)

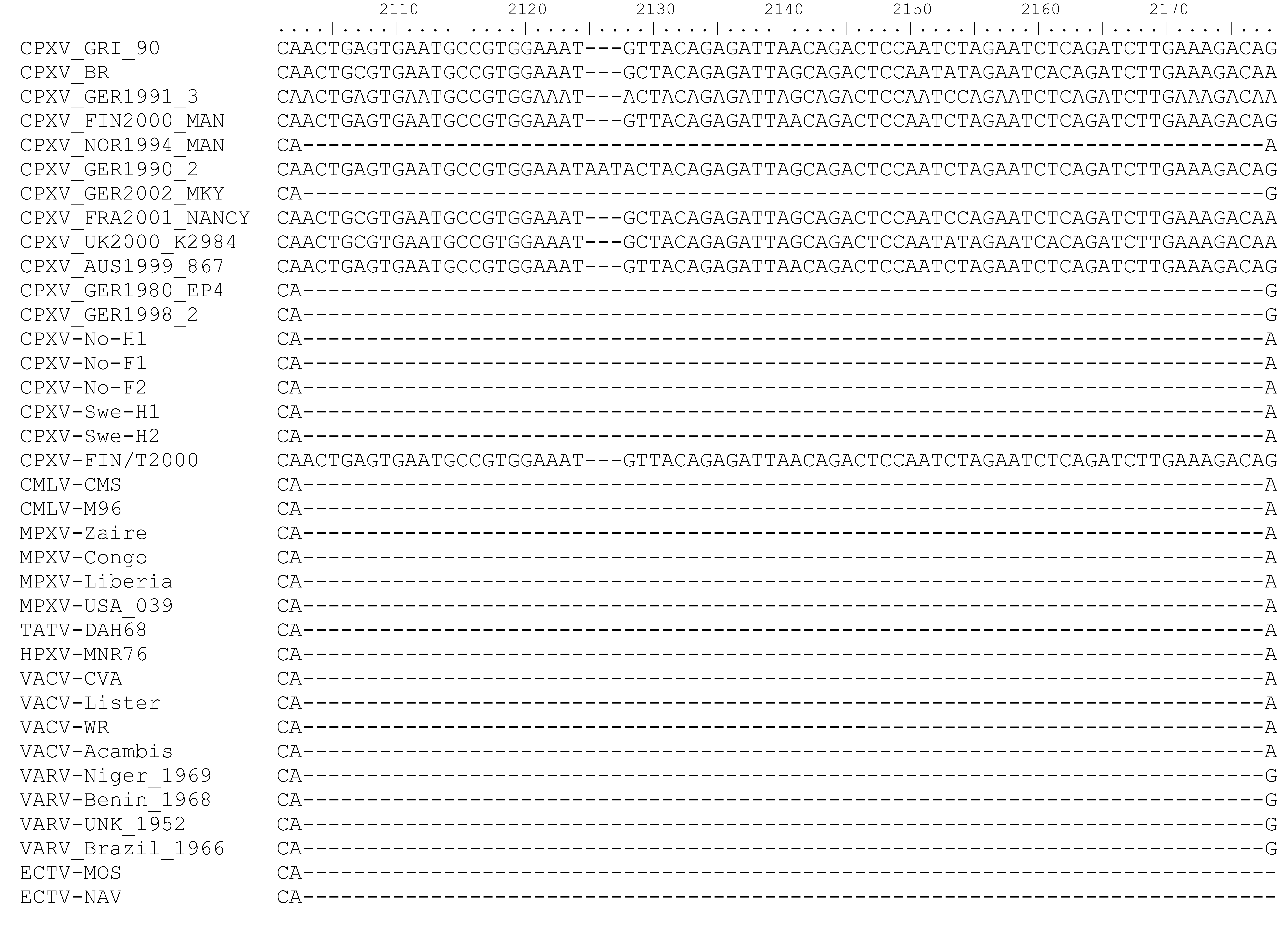

Supplement: Additional file 1 — Multiple sequence alignment of the DNA sequence of atip gene. Only regions of the alignment depicting the 72 bp deletion in some isolates (compared to that of CPXV-BR) are shown. [file 1743-422X-11-119-S1.tiff]

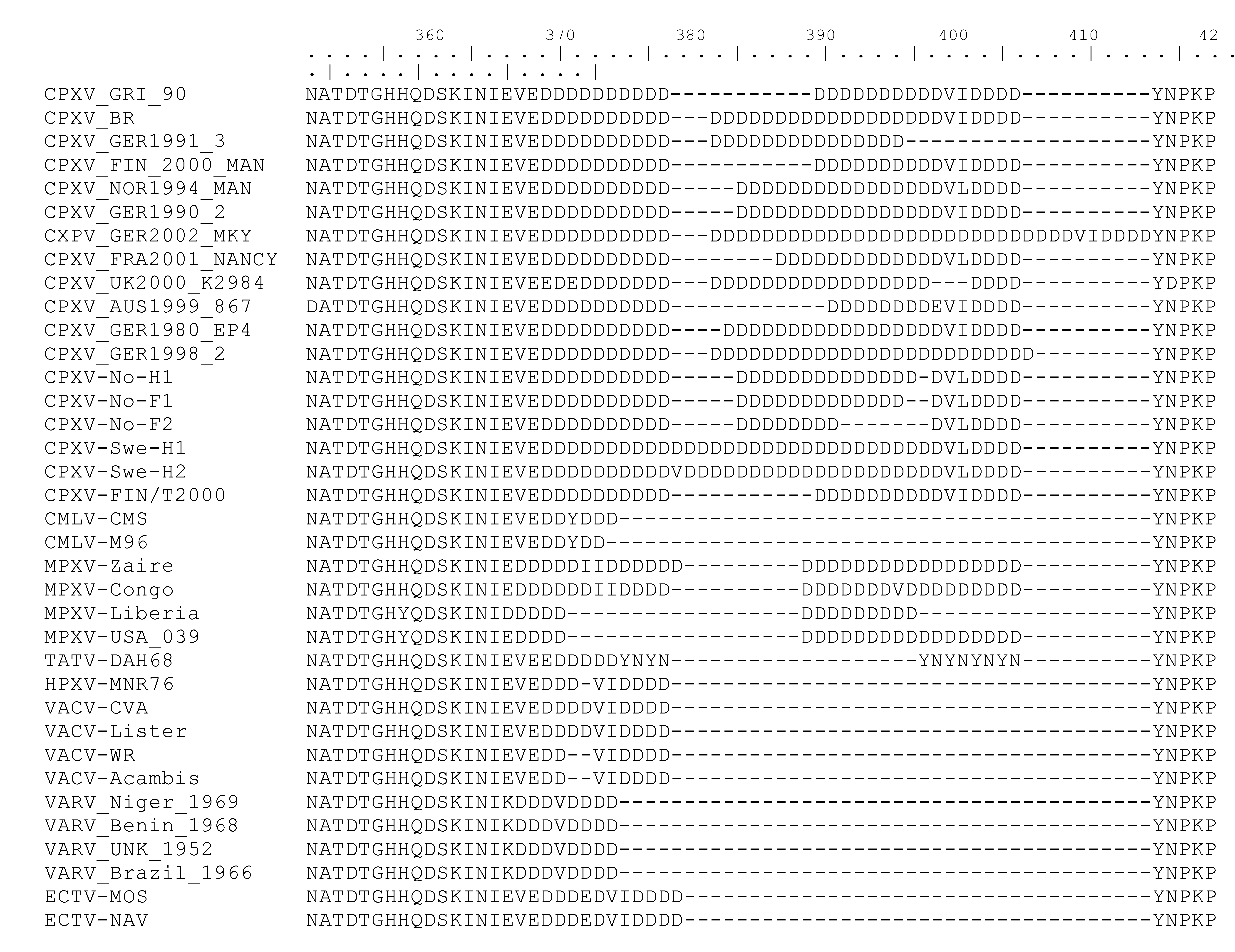

Supplement: Additional file 2 — Multiple sequence alignment of the amino acid sequence of the P4c protein. Only the region encompassing the C-terminal polyaspartate tract of the P4c protein of some strains of OPV is depicted. [file 1743-422X-11-119-S2.tiff]

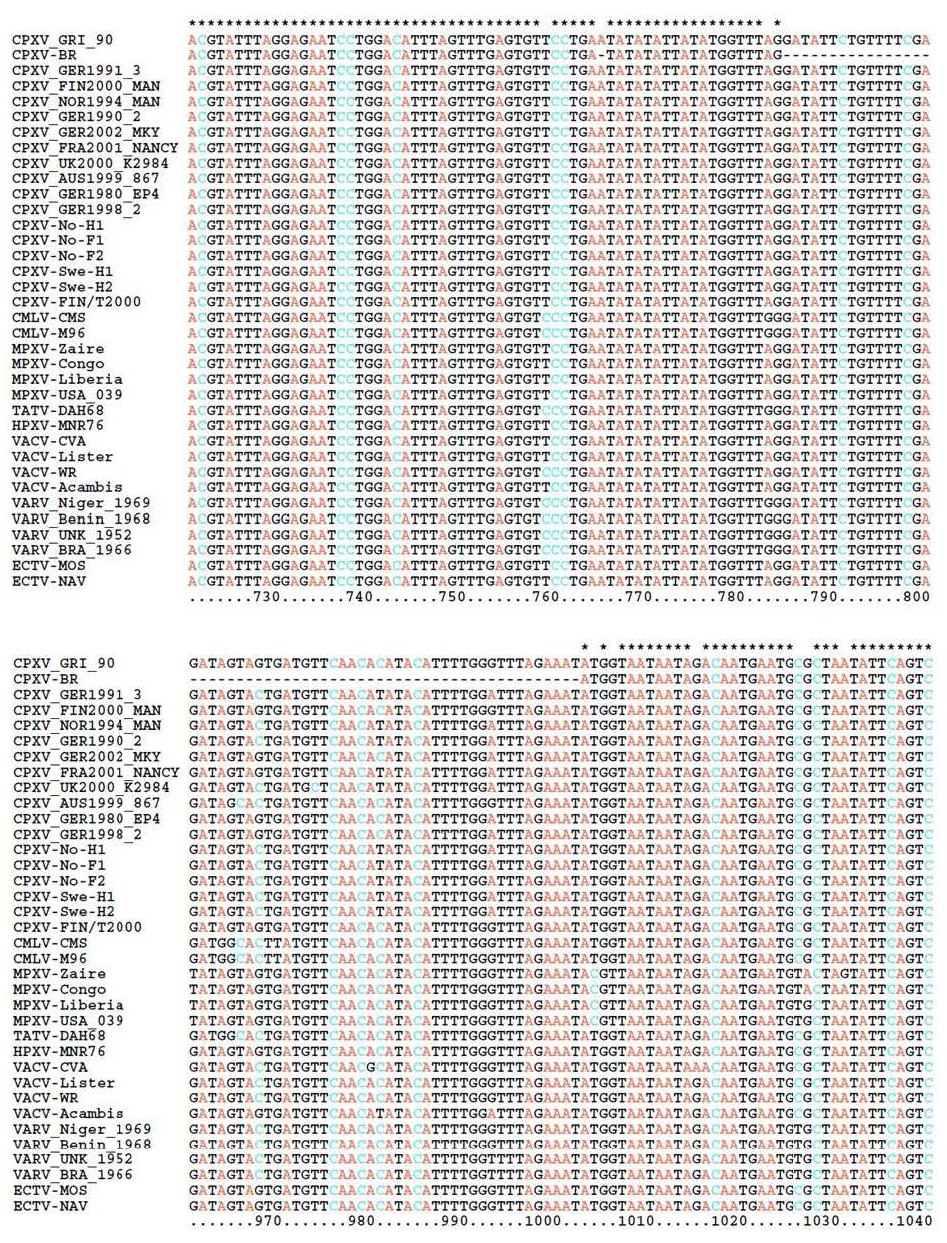

Supplement: Additional file 3 — Multiple sequence alignment of the DNA sequence of the p4c gene. Only the region showing the polymorphism that truncated the p4c gene of CPXV-BR is shown. Compared to the Fennoscandian CPXVs, the reference strain CPXV-BR has a single nucleotide deletion at position 765 which resulted in a frame shift mutation that introduced a stop codon at position 782–784. This terminated the first open reading frame (CPXV 161). The second open reading frame (CPXV 159) starts with the initiation codon at positions 1003–1005 of the alignment. [file 1743-422X-11-119-S3.tiff]

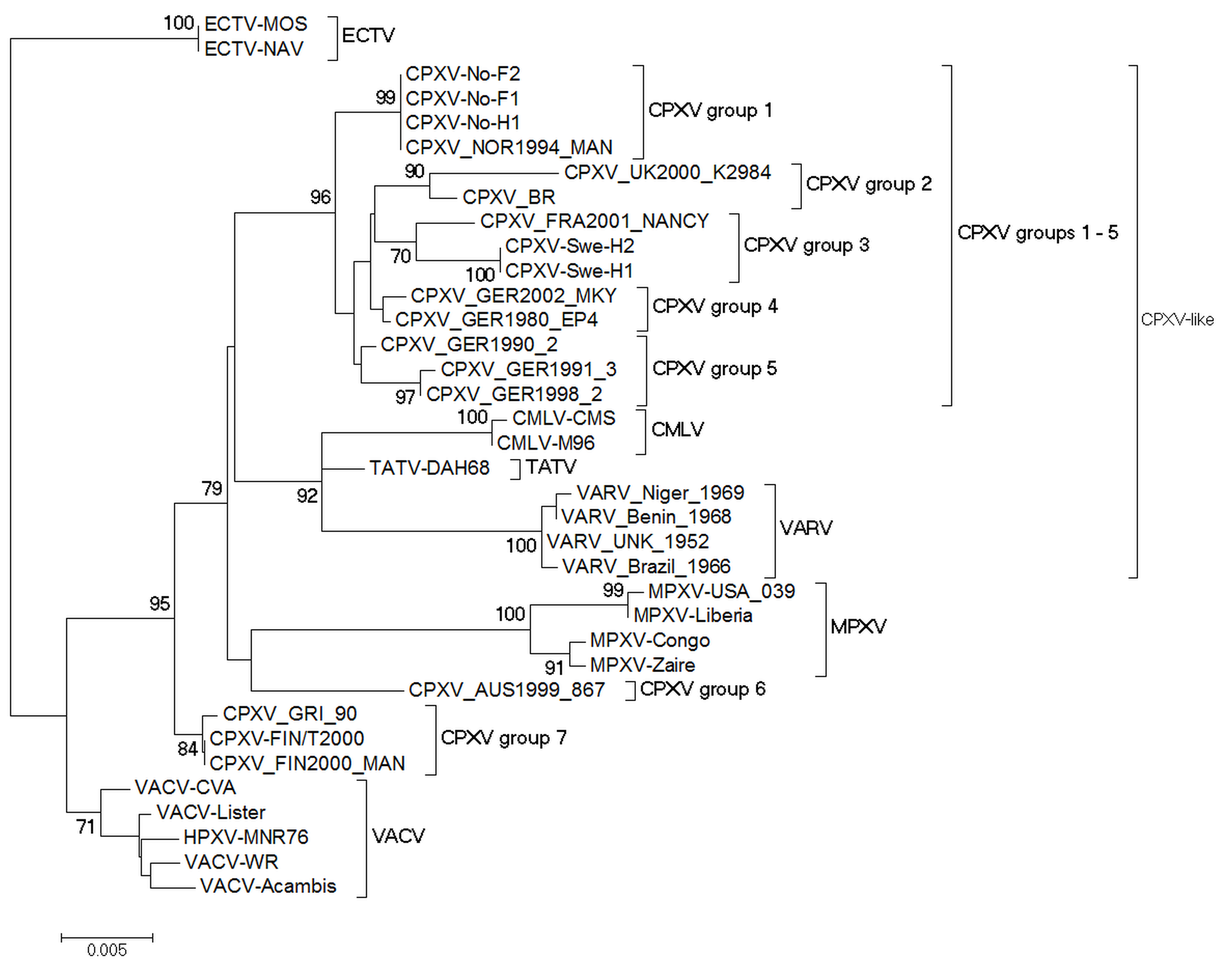

Supplement: Additional file 4 — Maximum Likelihood (ML) p4c gene phylogeny with ECTV as outgroup taxa. The ML tree was constructed with MEGA 5.0 as outlined in Methods. Bootstrap analysis with 1000 replicates was performed and only bootstrap values above 70% are shown. The scale represents substitutions per site. Neighbor Joining (NJ) tree and Bayesian Inference (BI) tree constructed with MEGA 5.0 and Mr Bayes 3.1.2 have tree topologies similar to the ML tree generated with MEGA 5.0. CPXVs (14 in total) belonging to clusters (groups) 1 to 5 are CPXV-like because the descended from the same common ancestor as the reference strain CPX-BR. CPXV groups 6 and 7 (4 viruses in total) are VACV-like because they are closer to VACV than CPXV-like viruses (groups 1 to 5). [file 1743-422X-11-119-S4.tiff]

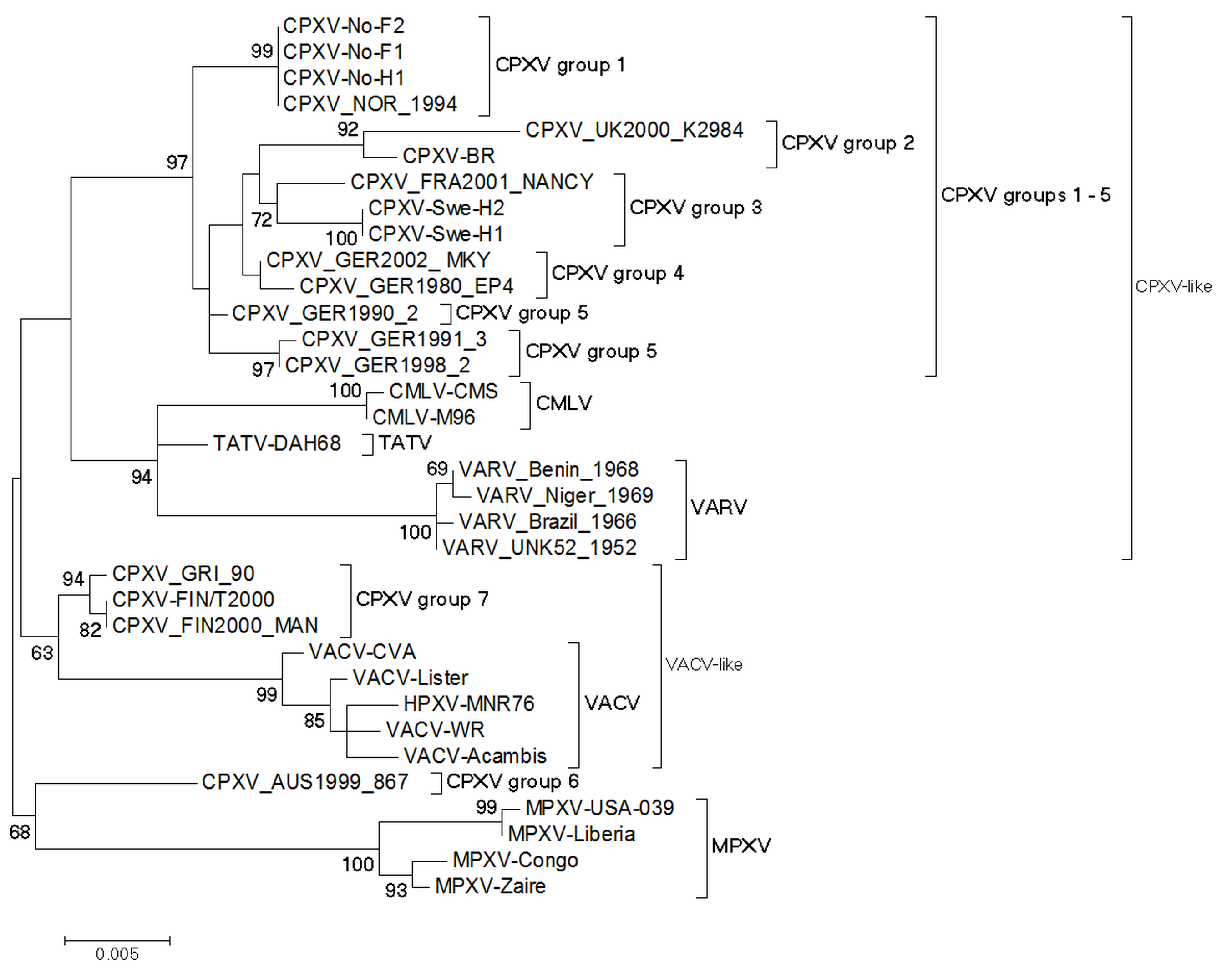

Supplement: Additional file 5 — Unrooted p4c gene phylogenetic tree constructed with Maximum Likelihood method. The ML tree was constructed with MEGA 5.05 as detailed in Methods. ECTV and CPXV-No-H2 sequences were excluded from the analysis. Bootstrap analysis with 1000 replicates was performed and only bootstrap values above 60% are shown. The scale represents substitutions per site. Neighbor Joining (NJ) tree and Bayesian Inference (BI) trees constructed with MEGA 5.05 and Mr Bayes 3.1.2 have tree topologies similar to the ML tree generated with MEGA 5.05. CPXVs (14 in total) belonging to clusters (groups) 1 to 5 are CPXV-like because they are grouped in the same clade with the reference strain CPXV-BR. CPXV groups 6 and 7 (4 viruses in total) are VACV-like because they are closer to VACV than CPXV-like viruses (groups 1 to 5). CPXV group 7 and VACV are grouped in the same clade. [file 1743-422X-11-119-S5.tiff]

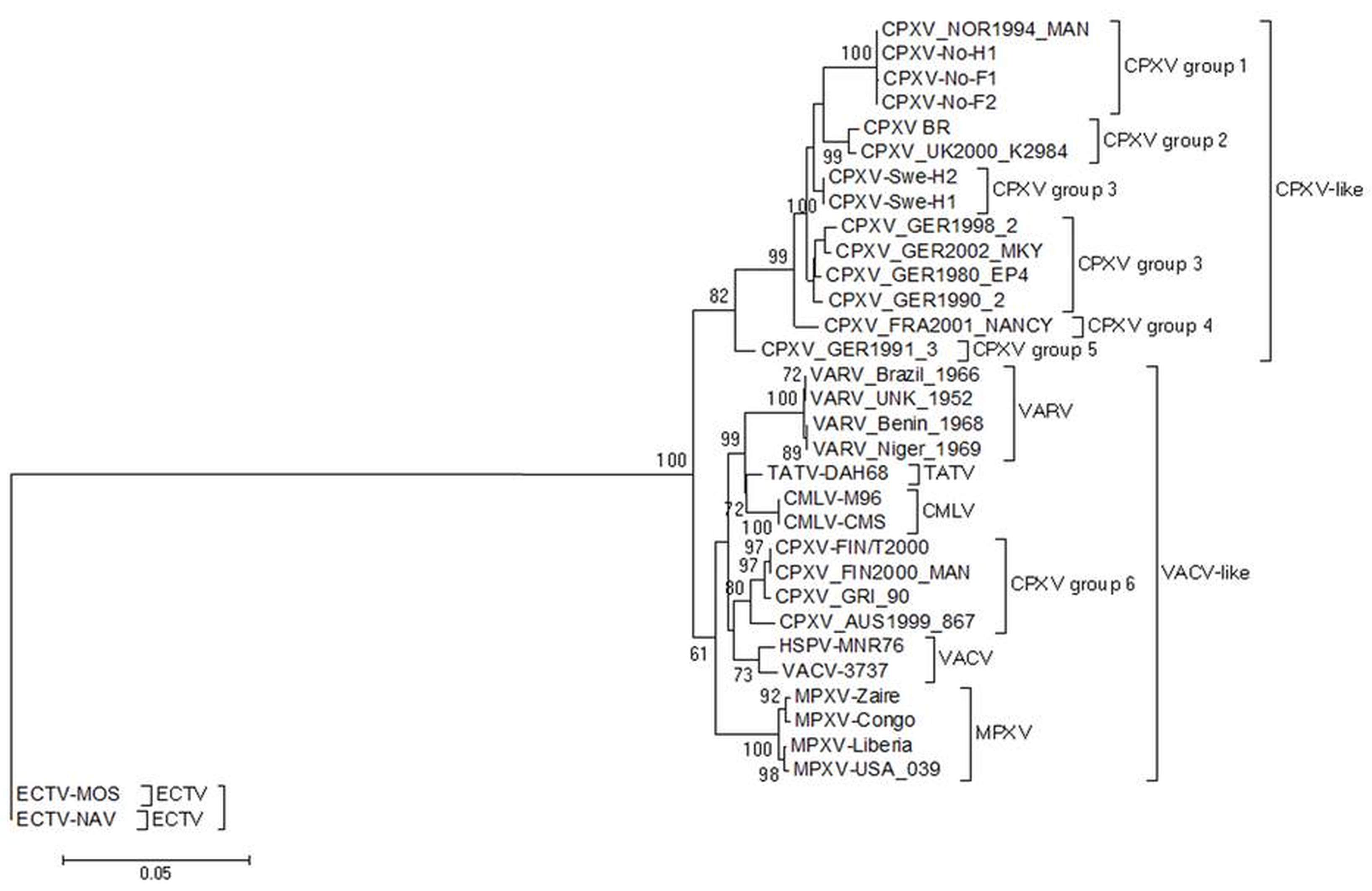

Supplement: Additional file 6 — The atip gene phylogenetic tree constructed with Maximum Likelihood in which vaccine and laboratory strains of vaccinia virus (VACV) were excluded. Laboratory and vaccine strains (as used in Figure 3) were excluded. Naturally isolated VACVs; HSPV-MNR76 and VACV-3737 (DQ377945) were the only VACV strains included for the construction of the phylogenetic tree. The ML tree was constructed with MEGA 5.05 as described in Methods. Bootstrap values were determined from 1000 replica sampling and only bootstrap values above 60% are shown. The scale indicates substitution per site. Neighbor Joining (NJ) tree constructed with MEGA 5.05 gave similar tree topology as the ML tree but with higher bootstrap support. The Bayesian Inference (BI) tree generated with Mr. Bayes 3.1.2 was poorly resolved. CPXVs (14 in total) belonging to clusters (groups) 1 to 5 are CPXV-like because the descended from the same common ancestor as the reference strain CPX-BR. CPXV group 6 are VACV-like because they are closer to VACV than to CPXV-like CPXVs and share a common ancestor with VACV. [file 1743-422X-11-119-S6.tiff]

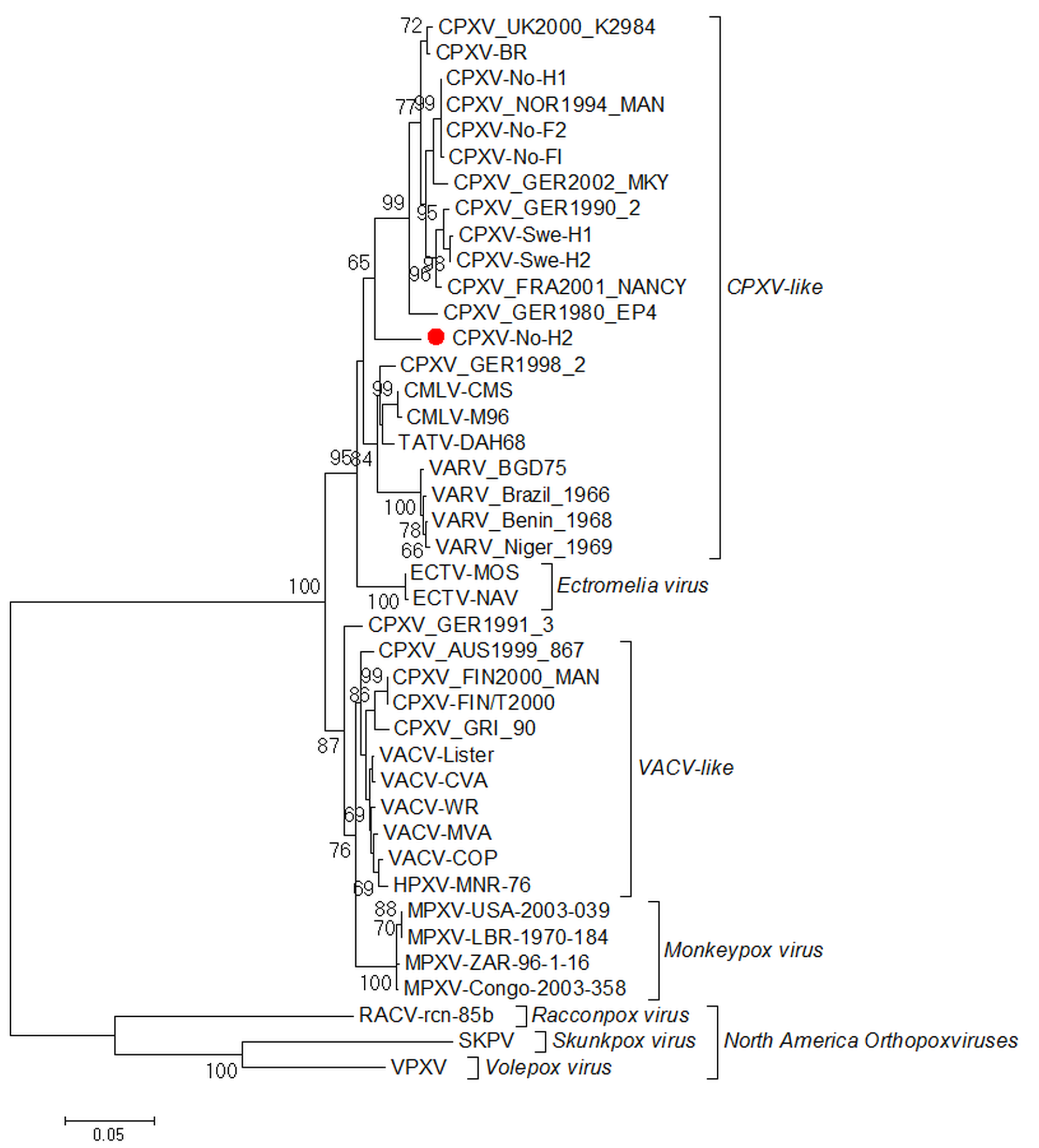

Supplement: Additional file 7 — Neighbor Joining (NJ) tree based on the nucleotide sequences of complete haemaggluttinin (HA) ORFs. The NJ tree was constructed from aligned sequences using MEGA 5.05. Bootstrap values were determined from 1000 replica sampling and only bootstrap values above 60% are shown. The bar indicates the divergence scale. The topology of the Maximum Likelihood (ML) tree constructed with MEGA 5.05 was similar to the NJ tree but with a lower bootstrap support in some of the nodes. [file 1743-422X-11-119-S7.tiff]
